# Characterizing Average Seasonal, Synoptic, and Finer Variability in Orbiting Carbon Observatory‐2 XCO_2_ Across North America and Adjacent Ocean Basins

**DOI:** 10.1029/2022JD036696

**Published:** 2023-01-27

**Authors:** Kayla A. Mitchell, Scott C. Doney, Gretchen Keppel‐Aleks

**Affiliations:** ^1^ Environmental Sciences University of Virginia Charlottesville VA USA; ^2^ Climate and Space Sciences and Engineering University of Michigan Ann Arbor MI USA

**Keywords:** OCO‐2, XCO_2_, carbon flux estimation, correlated error, synoptic variability, mesoscale variability

## Abstract

Variations in atmosphere total column‐mean CO_2_ (XCO_2_) collected by the National Aeronautics and Space Administration's Orbiting Carbon Observatory‐2 satellite can be used to constrain surface carbon fluxes if the influence of atmospheric transport and observation errors on the data is known and accounted for. Due to sparse validation data, the portions of fine‐scale variability in XCO_2_ driven by fluxes, transport, or retrieval errors remain uncertain, particularly over the ocean. To better understand these drivers, we characterize variability in OCO‐2 Level 2 version 10 XCO_2_ from the seasonal scale, synoptic‐scale (order of days, thousands of kilometers), and mesoscale (within‐day, hundreds of kilometers) for 10 biomes over North America and adjacent ocean basins. Seasonal and synoptic variations in XCO_2_ reflect real geophysical drivers (transport and fluxes), following large‐scale atmospheric circulation and the north‐south distribution of biosphere carbon uptake. In contrast, geostatistical analysis of mesoscale and finer variability shows that real signals are obscured by systematic biases across the domain. Spatial correlations in along‐track XCO_2_ are much shorter and spatially coherent variability is much larger in magnitude than can be attributed to fluxes or transport. We characterize random and coherent along‐track XCO_2_ variability in addition to quantifying uncertainty in XCO_2_ aggregates across typical lengths used in inverse modeling. Even over the ocean, correlated errors decrease the independence and increase uncertainty in XCO_2_. We discuss the utility of computing geostatistical parameters and demonstrate their importance for XCO_2_ science applications spanning from data reprocessing and algorithm development to error estimation and carbon flux inference.

## Key Points

- We attribute variability in XCO_2_ retrieved from NASA's OCO‐2 satellite to surface flux gradients, atmospheric transport, and error
- Seasonal and synoptic‐scale XCO_2_ variability reflects hemispheric and continental‐scale surface carbon flux gradients
- Correlated errors impart spatially coherent fine‐scale variability that significantly increases standard error in XCO_2_ aggregates

## 1 Introduction

Carbon flux accounting forms the basis of climate‐science applications that guide policy, track fossil fuel emissions, monitor the biosphere, and project global change. Spatiotemporal variations in atmospheric carbon dioxide, CO_2_, reflect the underlying uptake and release of CO_2_ by surface processes and atmospheric transport. Consequently, atmospheric CO_2_ measurements can provide a top‐down constraint for carbon flux inference, given that the signal from surface fluxes is disentangled from transport‐induced variability. Top‐down flux inference, achieved through atmospheric inverse modeling, requires a quantitative description of transport‐induced variability and error in CO_2_ measurements across a wide range of spatial and temporal scales. While there is currently good confidence in surface carbon fluxes estimated from inversion studies on hemispheric and annual scales, there is still disagreement in the corresponding surface fluxes on sub‐annual and regional scales (Baker et al., 2006; Gaubert et al., 2019; Gurney et al., 2002; Peiro et al., 2022). Uncertainties arise due to the limited spatiotemporal coverage of observations, differing model representations of atmospheric transport and mixing, and poorly constrained regional surface flux heterogeneity. In the past decade, space‐based instruments such as the National Aeronautics and Space Administration's Orbiting Carbon Observatory‐2 (NASA OCO‐2) satellite have provided a more complete global picture of total column average atmospheric CO_2_, XCO_2_ (Eldering et al., 2017).

Since its launch in July 2014, OCO‐2 has measured XCO_2_ with a high level of precision (between 0.1% and 0.5% or ∼1 ppm error per individual sounding) (O’Dell et al., 2018; Wunch et al., 2017) capable of reducing uncertainties in regional carbon flux inference (Miller et al., 2007; Rayner & O’Brien, 2001). Because XCO_2_ captures the CO_2_ abundance throughout a total atmospheric column, including the planetary boundary layer and free troposphere, it is less sensitive to vertical mixing and entrainment than measurements made near the surface. This mediates the uncertainties in transport models that arise when representing small‐scale vertical mixing and ties XCO_2_ more directly to surface fluxes via mass balance (Basu et al., 2018; Olsen & Randerson, 2004; Rayner & O’Brien, 2001). However, XCO_2_ is sensitive to rapid horizontal transport in the free troposphere, giving these measurements a large footprint that reflects large‐scale flux patterns more than local processes (Keppel‐Aleks et al., 2011). Atmospheric inversions of XCO_2_ can thus constrain surface fluxes at regional and continental spatial scales, bridging the gap between small‐scale direct flux measurements, which must be extrapolated to other areas, and global constraints, which cannot capture regional dynamics.

Transport‐induced XCO_2_ variability arising from the mixing of XCO_2_ concentration gradients must be resolved in inverse model frameworks to reveal surface flux information. The influence of transport on XCO_2_ variability has been well‐documented in time‐series such as that from the Total Carbon Column Observing Network (TCCON) (Wunch et al., 2011). TCCON is a network of ground‐based spectrometers with coverage that is more temporally dense and spatially sparse in comparison to space‐based XCO_2_ observing instruments. On sub‐seasonal scales, the most significant variations in simulated XCO_2_ are driven by synoptic‐scale advection (occurring over thousands of kilometers and lasting a few days to weeks) of continental‐scale spatial XCO_2_ gradients, as opposed to local flux variability (Keppel‐Aleks et al., 2011). Specifically, local fluxes are not the dominant influence on TCCON XCO_2_ variability, even on diurnal timescales. Sub‐seasonal variations in midlatitude TCCON XCO_2_ are primarily driven by synoptic‐scale advection across the hemispheric summertime north‐south gradient in XCO_2_, shaped by the mean distribution of growing season biosphere carbon uptake (Keppel‐Aleks et al., 2012). Synoptic‐scale TCCON XCO_2_ variability could reach up to half the peak‐to‐trough amplitude of the seasonal cycle. XCO_2_ variability at midlatitude TCCON sites differed during the summer based on the strength of the north‐south gradient in the area (Keppel‐Aleks et al., 2012). Outside of summer months, synoptic XCO_2_ variability is more similar across midlatitude TCCON sites when the gradient is weaker. Significant sub‐seasonal variability in TCCON XCO_2_ is also attributed to advection by mesoscale weather systems (occurring over ∼10s of km and lasting 1 day or less). Mesoscale variability at TCCON sites is typically between 0.2 and 0.5 ppm, 30%–50% of the magnitude of synoptic‐scale variability (Torres et al., 2019). The observed mesoscale variability was about half the magnitude of diurnal fluxes at Northern Hemisphere midlatitude TCCON sites but could be greater in magnitude than diurnal variability outside the growing season.

The temporal duration of individual OCO‐2 overpasses is too short and the repeat cycle of OCO‐2 orbits is too long to sample synoptic or mesoscale systems' time‐variability directly. The satellite has a repeat cycle of 16 days, acquiring at each time step a narrow swath of up to eight cross‐track samples that have individual spatial footprints of 2.4 km along‐track by 1.25 km cross‐track. While synoptic‐scale atmospheric transport is often explicitly resolved in inversion techniques, simulation of mesoscale transport is less common and errors/gaps in coverage inhibit OCO‐2 XCO_2_'s ability to capture real local gradients. For instance, clouds that obscure OCO‐2 measurements are often present in mesoscale weather systems. Some inverse frameworks have improved the spatial resolution of transport models to simulate mesoscale atmospheric transport despite the great required computational expense (Wesloh et al., 2020), but inversions on this scale require accurate representations of subgrid‐scale spatially coherent variability in assimilated XCO_2_.

To verify fine‐scale variability in OCO‐2 XCO_2_, recent studies have compared observed variability with simulated XCO_2_ or high‐resolution validation XCO_2_ collected from in‐situ sites or aircraft. Torres et al. (2019) used space for time substitution to characterize the influence of mesoscale transport on OCO‐2 v8 XCO_2_ by comparing high‐pass‐filtered (<250 km) along‐track spatial XCO_2_ variations to temporal mesoscale variations in TCCON XCO_2_. They observed greater spatially coherent along‐track variability in OCO‐2 XCO_2_ than what could be attributed to mesoscale transport (∼0.4 ppm along 250 km of orbit track). Combined with correlation length scales much shorter (∼10–30 km) than those associated with mesoscale systems, they concluded systematic bias contributes significant along‐track spatially coherent variability to OCO‐2 v8 XCO_2_. Baker et al. (2022) found similar OCO‐2 v10 XCO_2_ error correlation length scales of 20 and ∼10 km, noting the two distinct length scales that fit much of the data may be driven by different sources of error (fast‐changing errors related to surface parameters vs. slow‐changing errors related to atmospheric parameters). Bell et al. (2020) compared along‐track OCO‐2 v9 XCO_2_ variations with aircraft underflights equipped with a Multifunctional Fiber Laser Lidar (MFLL). They found agreement between OCO‐2 and MFLL on synoptic scales but disagreement on local scales (0.35 correlation with MFLL), supporting the finding that systematic errors contribute significant spatially coherent non‐transport structures at fine scales in OCO‐2 XCO_2_. Worden et al. (2017) used the NASA GMAO high‐resolution free‐running GEOS‐5 CO2 simulation to estimate natural fine‐scale variability in XCO_2_ (owing to wind or fluxes) and compared that to the observed variability in OCO‐2 V7 XCO_2_ occurring along 100 km of orbit track. They found larger observed variability than simulated natural variability occurring over that small ∼100 km neighborhood (simulated variability was ∼0.1 ppm and observed variability was ∼1.28 ppm). These studies have shown real signals driven by mesoscale transport or fluxes are entangled with fine‐scale correlated errors in OCO‐2 XCO_2_. Fine‐scale variability and correlations must be explicitly represented in inverse model frameworks or used to inflate observation error estimates. Model misrepresentation of subgrid‐scale variability can impart errors in inverted fluxes on urban to global scales (Chevallier, 2007; Corbin et al., 2008; Lauvaux et al., 2016).

The effect of spatially coherent biases on inverted flux uncertainty is largely dependent on the spatial and temporal scale of the bias and aggregation scheme. When assimilating XCO_2_ into inversions, soundings are often averaged over some distance of orbit track, typically close to the length of a model grid cell (e.g., ∼110 km for a 1° × 1° grid). Standard error estimates of the aggregated data are then used to evaluate model biases. For example, Hu et al. (2020) evaluated biases in monthly mean high‐resolution WRF‐VPRM model‐simulated XCO_2_ to time‐matched OCO‐2 v9 XCO_2_ data pairs aggregated in 1° × 1° grid boxes. Dong et al. (2021) used OCO‐2 v9 data integrated onto a weather‐biosphere‐online‐coupled model WRF‐Chem and CarbonTracker 2019 grids (20 km grid and 1° × 1° grids, respectively) for validation of simulated XCO_2_. Byrne et al. (2021) used OCO‐2 v10 XCO_2_ to optimize fluxes from the NASA Carbon Monitoring System‐Flux (CMS‐Flux) inversion at 2° × 2.5° spatial resolution. In the OCO‐2 v9 Model Intercomparison Project (MIP), XCO_2_ was averaged along 10 s spans of orbit track (∼70 km) before assimilation into the inverse model, assuming errors were not correlated within the 10 s span (Peiro et al., 2022). XCO_2_ The assumptions made about the data and employed in bias correction are made due to the long decorrelation length of atmospheric CO_2_ (500–1,000 km) (Chevallier, 2007). However, observed XCO_2_ correlation lengths are much shorter than these typical averaging lengths, resulting in correlated groups of data and error within the aggregate (Baker et al., 2022; Torres et al., 2019). Making false assumptions about the independence of each along‐track XCO_2_ sounding and its associated error leads to overconfidence in the XCO_2_ and incorrect error reductions (Baker et al., 2010).

Recent studies have tried to address fine‐scale error correlations to varying degrees, but challenges remain in representing and attributing the uncertainty they produce in inverted fluxes. Intermediate averaging, such as averaging 1 s or 2 s spans before averaging the full 10 s span, was tried in the v7 MIP (Crowell et al., 2019) and shown to improve aggregate error estimates (Baker et al., 2022). Using Lidar MFLL underflight validation data, Baker et al. (2022) evaluated flux errors that arise from representing measurement and error correlations in v10 XCO_2_. They employed an Observing System Simulation Experiment (OSSE) and found retrieval biases were much larger and more variable than parameterized biases and derived a 1D error estimation model that represented correlations between the data as exponentially decaying. The error model showed improvement upon constant correlation models such as that used in the V9 MIP, which set constant correlation coefficients of +0.3 for adjacent land retrievals and doubled this value to +0.6 for adjacent ocean retrievals. While the constant correlation model proved to be sufficient, the correlation coefficients are somewhat arbitrary. Baker et al. (2022) also used this twice‐the‐land relationship to double correlations over the ocean in their model because they did not have MFLL data over the ocean. Due to lacking validation data and assumptions that XCO_2_ statistics over the ocean should be fairly uniform, XCO_2_ correlation lengths over the ocean have typically been approximated using correlations that have been better characterized in retrievals over land. Correlations in OCO‐2 XCO_2_ imparted by systematic bias have not been explicitly studied to the extent needed to represent aggregate uncertainty in flux inversions.

Identifying sources of error in OCO‐2 XCO_2_ and correcting systematic biases is an ongoing effort. In bias correction, systematic error in XCO_2_ that correlates with retrieval parameters (e.g., aerosol quantities, albedo, or surface pressure) is corrected using multivariate regression. Improvements in the retrieval algorithm and parametric bias correction reduce these biases with each data release (Kiel et al., 2019; Kulawik et al., 2019; O’Dell et al., 2018; Wunch et al., 2017). Wunch et al. (2017) found generally good agreement with v7 XCO_2_ and TCCON validation data at global scales (RMS differences less than 1.5 ppm) but noted that significant spurious variability remains on local scales. Residual biases are greater above 45°N, over areas subject to pathlength errors due to scattering from clouds or aerosols, and over areas where errors in assumed surface pressure arise due to rough topography (Wunch et al., 2017). Erroneous surface pressure estimates can also occur in the meteorological reanalysis used in bias correction when sampled at incorrect times or if there are small misspecifications of instrument pointing, particularly over regions with rough topography (Kiel et al., 2019). Despite improvements in the retrieval algorithm, systematic biases over regional and finer scales in the latest version (v10) XCO_2_ can be large enough to impede surface flux estimation. Rastogi et al. (2021) compared bias‐corrected v10 XCO_2_ retrievals with in situ data‐constrained simulated XCO_2_ over North America. They found differences between the retrieved and simulated quantities on local scales (tens of kilometers) of the same magnitude as the imprint of surface fluxes in the total column and were able to attribute these differences to persisting fine‐scale systematic errors in XCO_2_. Error analysis and uncertainty quantification remain areas of active research that strive toward reaching the level of accuracy and precision required for XCO_2_ measurements to detect exceptionally subtle flux‐driven variations in the atmospheric column.

Until the variance budget is fully resolved and applied within inverse modeling frameworks, the representation of aggregated OCO‐2 XCO_2_ will cause large uncertainties in inverted fluxes on regional and sub‐seasonal scales. To understand the influence of atmospheric transport, surface processes, and error on different spatial and temporal scales, we characterize variability in OCO‐2 v10 XCO_2_ over North America and adjacent ocean basins. We evaluate spatial patterns in seasonal and synoptic‐scale variability that illustrate the relative impact of atmospheric circulation and surface flux gradients on XCO_2_ on different scales. On mesoscale and finer scales, we conduct an along‐track geostatistical analysis of variability to reveal possible retrieval errors and improve the representation of aggregated XCO_2_ and associated uncertainty in inverse frameworks. Relationships between variability and season, surface type, and pointing mode help narrow down the specific processes driving real and spurious XCO_2_ variability. Our analysis provides insight into both the dynamics of atmospheric CO_2_ and the applications and limitations of XCO_2_ measurements.

## 2 Materials and Methods

### 2.1 Characterizing Seasonal and Sub‐Seasonal Variability in XCO_2_

We use the OCO‐2 Lite Level 2 data product, which provides geolocated, bias‐corrected XCO_2_ aggregated into daily files (OCO‐2 Science Team/Michael Gunson, Annmarie Eldering, 2020). OCO‐2 spectrometers collect 24 spectra per second and yield over 100,000 XCO_2_ observations each day, about 10% of which are sufficiently cloud‐free scenes and have the precision required for scientific applications. We include all XCO_2_ soundings marked with a “good” quality warning flag from September 2014 to December 2019 and spanning between 180°–30°W and 14°–89° N in our analysis using MATLAB (2022) to process the data. This study domain encompasses North America and extends into the adjacent Pacific and Atlantic Ocean basins. XCO_2_ is derived from version 10 (V10) of the Atmospheric Carbon Observations from Space (ACOS) retrieval algorithm (O’Dell et al., 2012, 2018), and results include soundings collected in glint and nadir observation modes. We characterize average seasonal and sub‐seasonal variability in XCO_2_ within bins spanning 5° latitude and longitude. Results are compared by observation mode, season, and biome in Section 3. We use a TransCom regional mask that divides the domain into boreal, temperate, and tropical regions of N.A., the North Pacific, and the North Atlantic, publicly available by the current OCO‐2 V10 MIP (Figure S1 in Supporting Information S1). Across the study domain, there are about ∼500,000–700,000 observations per month, with fewer observations (∼300,000–500,000) in December, January, and February.

**Figure 1:**
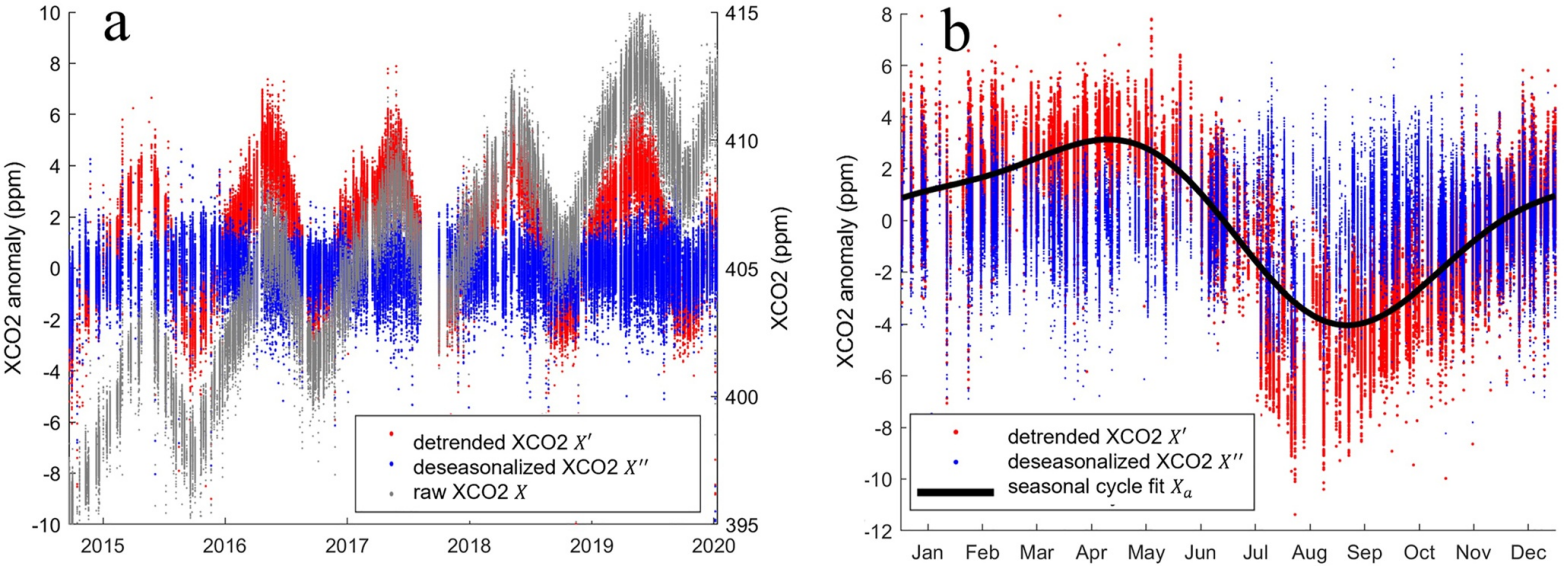
For each 5° bin, raw XCO_2_ is detrended to compute a series of anomalies. For a bin over Hawai'i, we show (a) time series of raw XCO_2_, annual $X^{\prime }$ and sub‐seasonal $X^{\prime \prime }$ XCO_2_ anomalies. (b) $X^{\prime \prime }$ are computed by removing the spatial bin means $\bar{X}$, the low‐pass filter representing interannual variability $X_{lp}$, and the mean annual cycle of annual XCO_2_ anomalies $X_{a}$. Note: (b) shows the full XCO_2_ time series grouped by month, not the average over all years. Data density was lower earlier in the mission (fewer than 500,000 observations per month in 2014 and 2015) as sampling patterns, decontamination cycling, calibration, and ground station communication were being optimized. Starting in the summer of 2015, the OCO‐2 team employed the V7/7r algorithm to reprocess the data record and develop corrections for these different issues, as summarized in Crisp et al. (2017). The large gap in observations spanning late July 2017 through September 2017 occurred due to band tracking and potentiometer issues, leading to an instrument reboot and extended period during which XCO_2_ data was not created or invalid.

We compute a series of anomalies to characterize v10 XCO_2_ variability on seasonal and sub‐seasonal scales (Figure 1a). First, we detrend the long‐term anthropogenic temporal increase in XCO_2_ using a linear regression computed on the time series of all XCO_2_ in the domain ($\bar{X})$. We subtract the long‐term temporal trend $\bar{X}$ from the XCO_2_ time series and remove the detrended mean of each box 〈X〉 from the corresponding XCO_2_ in Equation 1.

(1) $$X^{\prime }=X_{raw}-\bar{X}-\langle X\rangle$$

From the resulting detrended, spatial annual anomalies (denoted as $X^{\prime }$), we compute the average seasonal cycle for each bin in Equation 2 (Figure 1b). We fit the mean annual cycle of each bin with a first and second harmonic ($X_{a}$). Average seasonal amplitudes for each bin are computed as the peak‐to‐trough difference of $X_{a}$. To account for additional interannual variation, we compute a 6‐month low‐pass filter on annual anomalies ($X_{lp}$). Sub‐seasonal XCO_2_ anomalies, $X^{\prime \prime }$, are calculated by removing the annual cycle and low‐pass filter from $X^{\prime }$ (Equation 3).

(2) $$X_{a}=X^{\prime }\left(t,b\right)=b_{1}\;\sin\left(2\pi \left(t+b_{2}\right)\right)+b_{3}\;\sin\left(4\pi \left(\;t+b_{4}\right)\right)$$

(3) $$X^{\prime \prime }=X^{\prime }-X_{a}-X_{lp}$$

### 2.2 Geostatistical Analysis of Fine‐Scale Variability in XCO_2_

**Figure 2:**
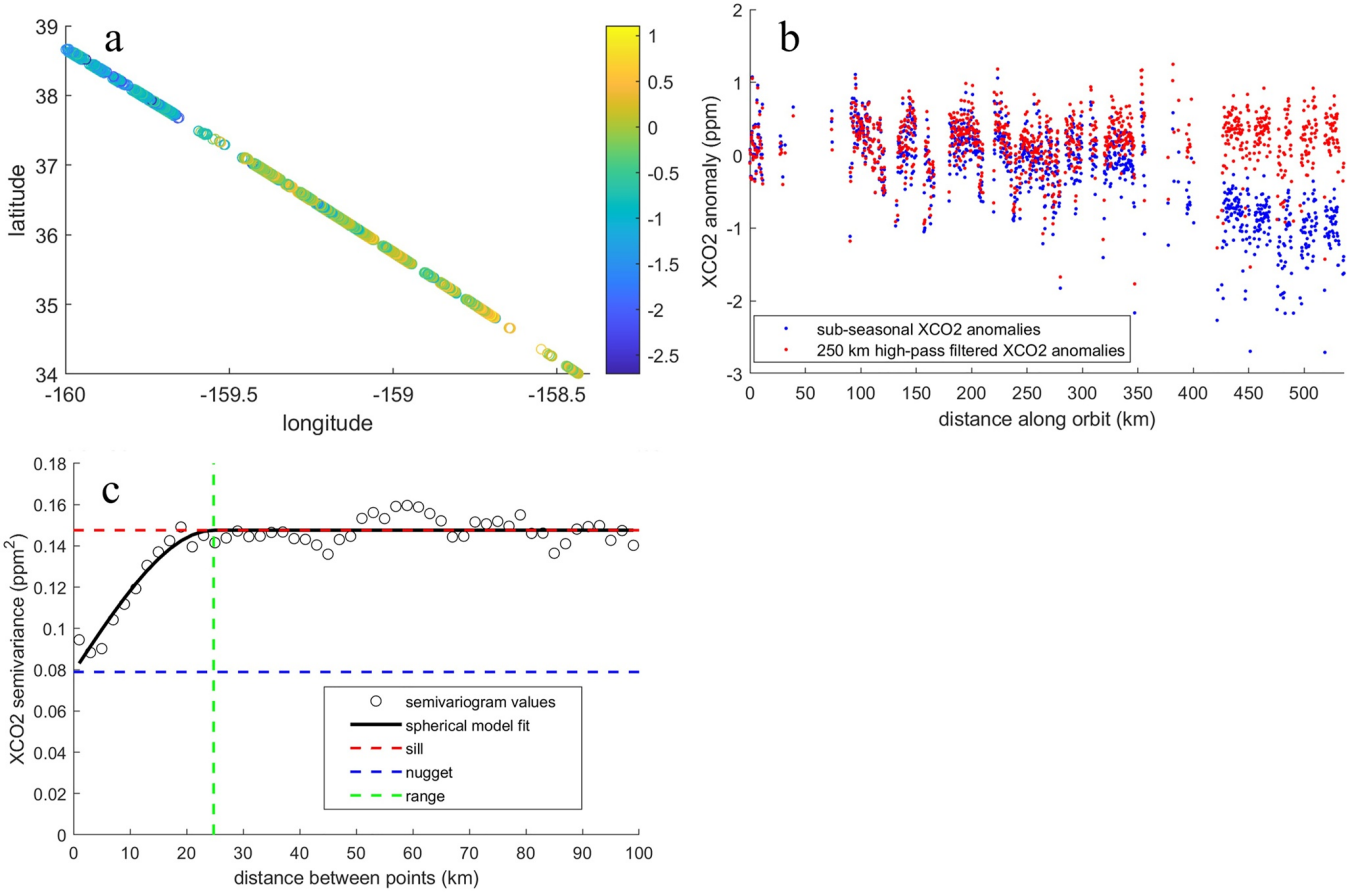
(a) Sub‐seasonal XCO_2_ anomalies $X^{\prime \prime }$ collected within a 5° bin during one orbit pass are (b) high pass filtered to remove variations occurring on spatial scales longer than 250 km to isolate mesoscale and finer variations. (c) The average semivariance of high‐pass filtered $X^{\prime \prime }$ anomalies for 50 lag distances between 0 and 100 km is measured by the experimental semivariogram and fit with a spherical model. The theoretical semivariogram estimates the total sample variance (sill, $c_{\infty }$), the random variance (nugget, $c_{0}$), the resolved variance (sill minus nugget, $c_{s}$), and the length at which two soundings become independent (range, $a_{space}$).

We use geostatistical methods to characterize the magnitude and spatial coherence of variability in XCO_2_ on two sub‐seasonal scales corresponding to synoptic and mesoscale atmospheric circulation because sub‐seasonal variability in XCO_2_ is largely driven by atmospheric transport. We divide sub‐seasonal variability into synoptic and finer scales as variability in $X^{\prime \prime }$ occurring on spatial scales longer and shorter than 250 km. Torres et al. (2019) demonstrated the 250 km spatial cutoff isolates mesoscale and finer variability in OCO‐2 XCO_2_ from synoptic‐scale variations. We compute a 250 km low‐pass filter on $X^{\prime \prime }$ along each orbit track (Figure 2a) using the spherical distance between two coordinates on Earth's surface as distance along orbit. To apply the filter, up to eight cross‐track soundings were centered onto a one‐dimensional track and gap‐filled using 1‐D linear interpolation on a spherical surface. Variations passed by the 250 km digital low‐pass filter are subtracted from XCO_2_ in their original position to compute fine‐scale XCO_2_ anomalies that capture variations on the atmospheric mesoscale (Figure 2b).

For each orbit pass through a 5° bin, we compute the experimental and theoretical semivariogram for fine‐scale XCO_2_ anomalies (Figure 2c). The experimental semivariogram, γ(h) (Equation 4), measures how related two points are to one another at different separation (lag, *h*) distances (Cressie & Hawkins, 1980). We compute the average semivariance for a total number of pairs N at 50 lag distances h centered between 0 and 100 km. An orbit has sufficient observations to compute the semivariogram if the spatial span of the retrievals in a 5° × 5° bin is at least 100 km along‐track and there are at least 50 good observations for each along‐track step (up to eight cross‐track footprints are retrieved for each along‐track step). $X_{k}$ and $X_{k+h}$ represent the fine‐scale XCO_2_ anomaly at locations k and k+h. We fit each experimental semivariogram with a spherical model (Equation 5) to compute the theoretical semivariogram (Figure 2c). The theoretical semivariogram estimates the total sample variance (sill, $c_{\infty }$), the random variance (nugget, $c_{0}$), the resolved variance (sill minus nugget, $c_{s}$), and the length at which two soundings become independent (range, $a_{space}$).

(4) $$\gamma \left(h\right)=\frac{1}{2N\left(h\right)}\sum_{k=1}^{N_{h}}{\left[X_{k}-\left(X_{k+h}\right)\right]}^{2}$$

(5) $$\gamma \left(h\right)=\left\{\begin{array}{c} c_{0}+\left(c_{\infty }-c_{0}\right)\left(\frac{3h}{2a_{space}}-\frac{1h^{3}}{2{a_{space}}^{3}}\right)\;for\;h\leq a_{space}\\ c_{\infty }\;for\;h>a_{space} \end{array}\right.$$

From all modeled parameter estimates and associated errors, we compute weighted averages of $c_{\infty }$, $c_{0}$, and $a_{space}$ for each bin (Equation 6) using two approaches. The variable x represents the modeled parameter estimate ($c_{\infty }$, $c_{0}$, or $a_{space}$) and the variable $\sigma^{x}$ represents error in estimated $c_{\infty }$, $c_{0}$, or $a_{space}$. Because errors scale with the magnitude of estimated parameters, we computed averages using the inverse of error (σ) as well as the proportionate error (σ/x). Weighted averages computed from the two approaches were only significantly different for range estimates (Section 3), and we present results computed using inverse error. Average spatially coherent fine‐scale variance, $\langle c_{s}\rangle$, is calculated in Equation 7 as the random variance subtracted from the average total variance.

(6) $$\langle a_{space}\rangle ,\;\langle c_{0}\rangle ,\;\langle c_{\infty }\rangle =\bar{x}=\frac{\sum_{i=1}^{N}x_{i}*\frac{1}{\sigma_{i}^{x}/x_{i}}}{\sum_{i=1}^{N}\frac{1}{\sigma_{i}^{x}/x_{i}}}$$

(7) $$\langle c_{s}\rangle =\langle c_{\infty }\rangle -\langle c_{0}\rangle$$

We use error estimates computed from the spherical model to assess the goodness of fit of each modeled parameter to the experimental semivariogram. If error for each estimated $c_{\infty }$, $c_{0}$, $a_{space}$ is larger than the value of the estimated parameter, we omit those poorest fits from the computation of the total bin averages. The majority of errors included $c_{\infty }$ were less than 10% of the parameter value, ∼10% of the estimated $c_{0}$ parameter, and less than 30% of the estimated $a_{space}$ parameter. Using inverse error to weight the parameters ensured that the results we present can be interpreted in good confidence because values with the best spherical model fits are given more weight than values with poorer spherical model fits.

Average synoptic‐scale variance for each bin is computed as the remainder of total sub‐seasonal variance after subtracting the average total fine‐scale variance $\langle c_{\infty }\rangle$. To compare variance by surface type, we repeat the geostatistical analysis using orbit passes over either majority land, water, or mixed surface types. We present our results in terms of variability, the square root of spatially coherent and random variance: ${\langle c_{\infty }\rangle }^{1/2}$, ${\langle c_{s}\rangle }^{1/2}$, and ${\langle c_{0}\rangle }^{1/2}$.

## 3 Results and Discussion

### 3.1 Mean Spatial XCO_2_ Anomalies

**Figure 3:**
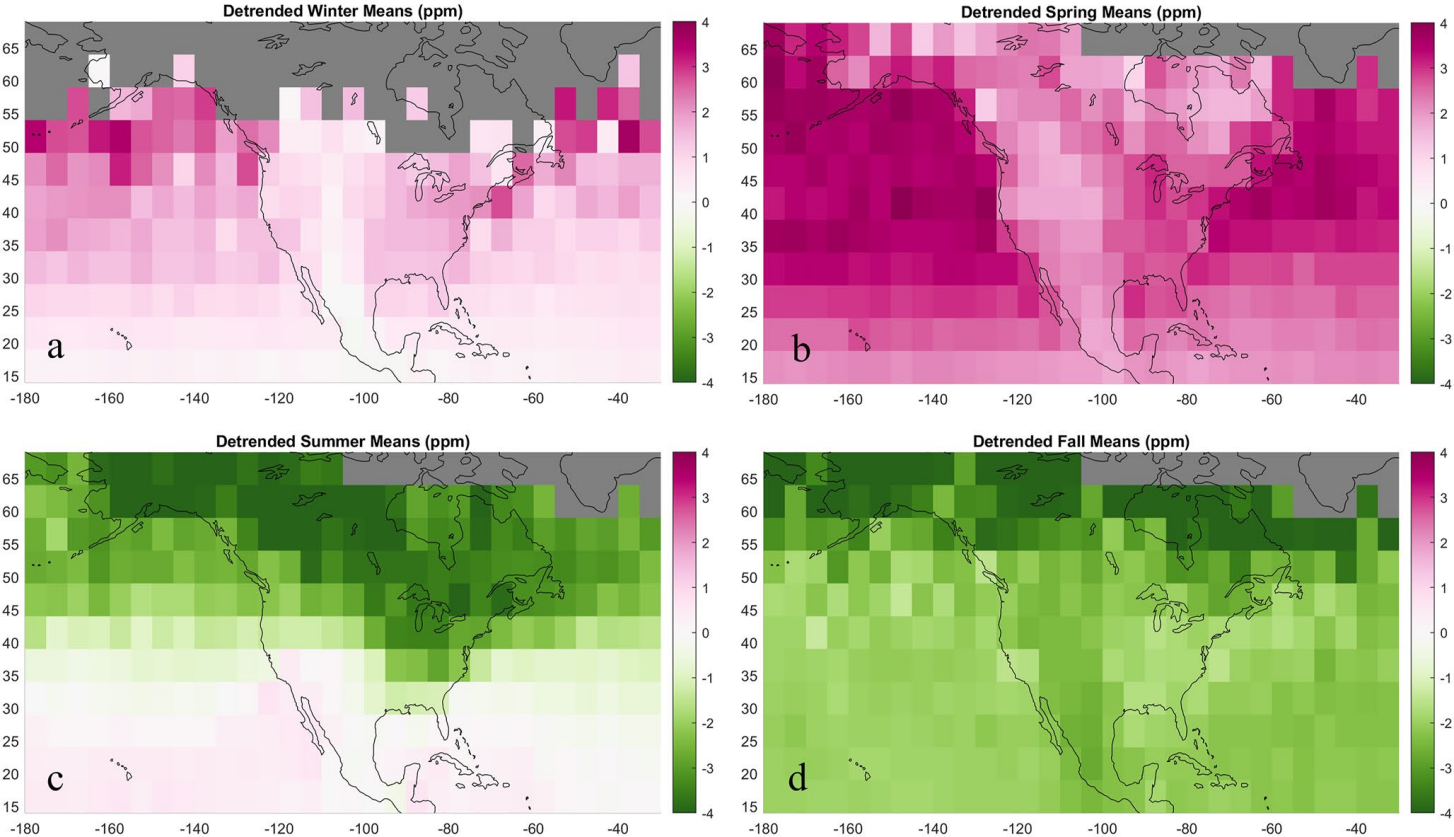
Long‐term temporally detrended anomaly averages for winter (a), spring (b), summer (c), and fall (d).

Mean spatial anomalies by season (Figure 3) indicate where average XCO_2_ concentrations were relatively enriched (positive) or depleted (negative) from 9‐2014 to 12‐2019 compared to the domain mean. During the summer (June, July, and August), there is a large increasing gradient of ∼4 ppm from north to south, centered around 39°N. Summertime anomalies exhibit the most pronounced gradient across all seasons and most closely follow mean zonal circulation. During the fall (September, October, and November), XCO_2_ anomalies across the domain are negative, with the most negative anomalies occurring above 54°N. There is an east‐west contrast across the continent where greater detrended XCO_2_ concentrations occur over the western United States and adjacent Atlantic Ocean and lower detrended XCO_2_ occur over the western United States and tropics. During the winter (December, January, and February), anomalies across the domain are positive and the same east‐west contrast is present (lower anomalies to the western United States and tropics and greater anomalies to the eastern United States). Over the ocean, there is a decreasing north‐south gradient in anomalies. Certain high latitude bins are omitted due to OCO‐2's wintertime data collection gaps. Anomalies are most positive during the spring (March, April, and May). The most negative anomalies occur over the boreal continental region (Table 1). The most positive anomalies occur over the northern Pacific temperate region. Average seasonal anomalies are compared by region in Table 1.

**Table 1:** Average XCO_2_ Anomaly by Season and Characteristics of Seasonal Variability Compared by Region

	Winter mean (ppm)	Spring mean (ppm)	Summer mean (ppm)	Fall mean (ppm)	Seasonal amplitude (ppm)	Seasonal maximum (decimal yr)	Seasonal minimum (decimal yr)
N. A. Boreal	1.0	2.3	−3.8	−3.7	9.8	0.36	0.66
N Pacific Temperate	1.5	3.3	−0.9	−2.1	7.6	0.36	0.72
W Pacific Tropical	0.2	2.1	0.5	−1.9	4.6	0.39	0.79
E Pacific Tropical	0.2	2.1	0.3	−2.0	4.9	0.39	0.78
N. A. Temperate	1.0	2.4	−1.8	−2.2	7.1	0.35	0.70
Northern Ocean	2.4	2.8	−3.0	−3.7	9.9	0.37	0.67
N Atlantic Temperate	1.0	2.9	−0.7	−2.1	6.8	0.36	0.73
Atlantic Tropical	0.3	2.1	0.2	−2.1	5.1	0.39	0.78
Tropical S.A.	0.2	2.1	0.2	−2.1	5.3	0.40	0.77
All	1.2	2.8	−1.6	−2.5	7.7	0.37	0.75

Mean seasonal anomalies exhibit an annually reversing north‐south gradient shaped by zonal circulation of north‐south distribution of surface fluxes. During the summer, there is greater carbon uptake by the terrestrial biosphere in higher latitudes, creating an increasing north‐south gradient bounded by the jet stream. During the winter, respiration outweighs photosynthesis and fossil fuel emissions are concentrated in higher latitudes, creating a decreasing north‐south gradient. Summertime anomalies follow dominant wind patterns and constant potential temperature surfaces at 700 hPa. Outside of summer months, east‐west contrasts over the continent suggest the influence of meridional flow. High‐velocity westerly winds travel south over the coastal Pacific adjacent to the west coast, diverting air away from the continent. The east‐west contrasts we observe could also be influenced by easterly trade winds deflecting off the North Pacific High, a semi‐permanent subtropical anticyclone, and circulating lower latitude air northward along the western continent. During the springtime, we observe the largest land‐ocean contrast in XCO_2_ at the west coast boundary (XCO_2_ over 2 ppm greater over the Pacific Ocean than immediately across the coastline). We expect patterns to reflect mean atmospheric patterns and the large‐scale north‐south carbon flux distribution rather than local underlying carbon fluxes due to rapid horizontal mixing in the free troposphere and XCO_2_'s large footprint. Consequently, the magnitude and sharp boundary of this land‐ocean difference are difficult to interpret, given that underlying fluxes over the continent are larger and more seasonally variable than those over the adjacent ocean. Ongoing discussion in the OCO‐2 community focuses on differences between land and ocean XCO_2_ observations. These results prompt investigation into whether the divergence of easterly and westerly winds, a land‐ocean retrieval bias, or systematic bias related to underlying surface properties are driving land‐ocean XCO_2_ differences across the west coast, particularly during spring months.

### 3.2 Seasonal Variability in XCO_2_

**Figure 4:**
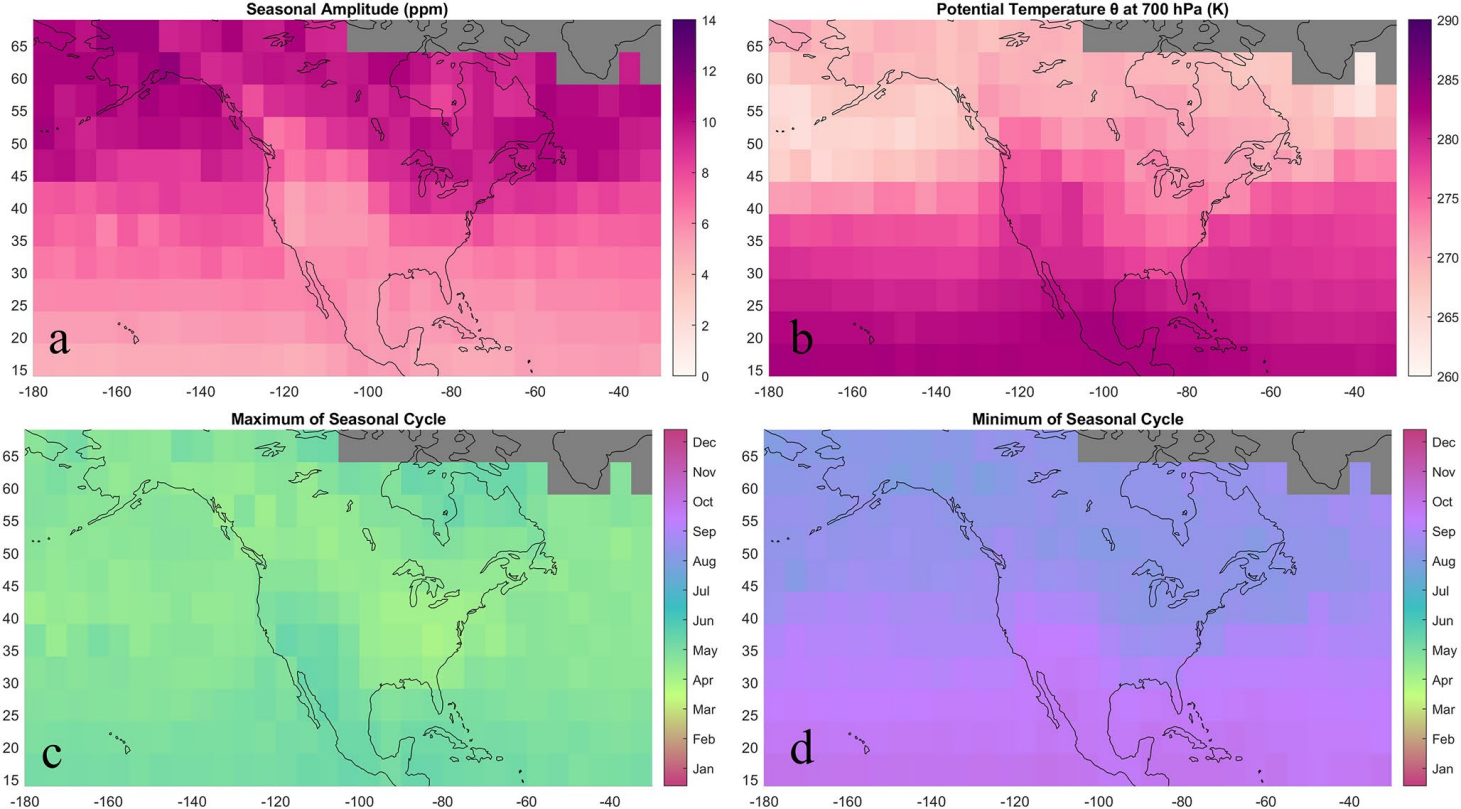
(a) Average peak‐to‐trough seasonal cycle amplitudes follow mean wind patterns and spatially correlate with changes in (b) average potential temperature at 700 hPa. The seasonal cycle reaches a maximum (c) between April and May and a minimum (d) between August and October. Bins with greater amplitudes generally reach an earlier seasonal cycle maximum and minimum.

We present the average peak‐to‐trough amplitude (Figure 4a) and phasing (Figures 4c and 4d) of the mean seasonal cycle in XCO_2_ ($X_{a}$). Seasonal amplitudes generally increase with latitude while also exhibiting substantial east‐west variation over the continent. The greatest amplitudes (reaching 11.5 ppm) are concentrated from the highest latitudes over the Northern Ocean and boreal continental region (Table 1) to a meandering southern boundary that follows the jet stream and gradient in potential temperature *θ* at 700 hPa, a dynamical tracer in the free troposphere computed using Poisson's equation and 700 mb temperatures provided in the OCO‐2 lite files (Figure 4b). Land and ocean tropical regions have the lowest amplitudes on average, forming a north‐south gradient of ∼5 ppm. Seasonal amplitudes also decrease on average from west to east, from the North Pacific Temperate region (7.6 ppm) to the continental Temperate region (7.1 ppm) to the North Atlantic Temperate region (6.8 ppm). The exception to these large‐scale patterns occurs over the western United States, which has the lowest amplitudes in the domain (under 5 ppm). A sharp land‐ocean contrast in amplitudes manifests across the western coastline; bins over the western continent have distinctly lower amplitudes (up to 4 ppm) than the adjacent bins over the coastal Pacific. This feature may be driven by transport, with greater and lesser potential temperature (Figure 4b) over the Pacific Northwest and adjacent Pacific Ocean, respectively. Alternatively, this feature may be caused by low biases in retrievals over the western continent and prompts further investigation.

Across the domain, the maximum in $X_{a}$ occurs between April and May (Figure 3c) and the minimum occurs between August and October (Figure 3d). Bins with greater amplitudes in Figure 3b tend to reach an earlier maximum and minimum than bins with lower amplitudes. The seasonal cycle in XCO_2_ lags behind that of surface fluxes due to the time required for surface fluxes to mix into the free troposphere, enabling OCO‐2 XCO_2_ to capture both extrema of the seasonal cycle even in many locations with wintertime data gaps. The root mean square error of $X_{a}$, representing average deviation from the average annual cycle fit, was 2.7 ppm on average and ranged from 0.5 to 4.4 ppm, scaling with amplitude.

Spatial patterns in $X_{a}$ seasonal amplitudes resemble average zonal circulation winds rather than patterns in underlying surface flux seasonality, supporting findings from Keppel‐Aleks et al. (2011) that the seasonal cycle in XCO_2_ is shaped by the large‐scale north‐south flux distribution. The spatial pattern we observe is consistent with XCO_2_ amplitudes modeled by Sweeney et al. (2015), who showed the high‐latitude feature of greater amplitudes extends across the globe. Areas such as the Arctic tundra with large seasonal amplitudes despite having small biospheric and anthropogenic fluxes are influenced by transported fluxes. Zonal transport of highly seasonal fluxes from boreal regions has been used to explain increasingly large seasonal cycles in column CO_2_ over the Arctic (Keppel‐Aleks et al., 2011, 2012; Olsen & Randerson, 2004). Sweeney et al. (2015) suggested that northward transport from lower latitudes, in addition to zonal transport of boreal fluxes, contributes to large seasonal cycle amplitudes observed in high latitudes. Western United States anomalies that depart from mean zonal circulation in Figure 3a suggest the influence of meridional transport along the western continent, which could carry northwestern U.S. fluxes and the imprint of their seasonality to higher latitudes. Average characteristics of seasonal variability are compared by region in Table 1.

### 3.3 Synoptic‐Scale Variability in XCO_2_

Average synoptic‐scale variability in XCO_2_, computed as average sub‐seasonal anomaly variability occurring on spatial scales longer than 250 km, and fine‐scale variability (<250 km) comprise total sub‐seasonal variability in XCO_2_ The components of fine‐scale variability are summarized in Table 2. In Table 3, synoptic variability and fine‐scale variability are compared by region. Average synoptic variability was greatest over the continental Boreal region, reaching a maximum of 1.5 ppm along the west coast of Canada. Over the continent, synoptic variability decreases on average from the Boreal region to the tropics, but a cluster of greater variability also occurs over eastern bins in the Temperate region. Over the ocean, synoptic variability exhibits more uniform latitudinal patterns and is greater (over 0.5 ppm) in middle and high latitudes. Synoptic variability was lowest (under 0.5 ppm) over the subtropical and tropical oceans.

**Table 2:** Description of Geostatistical Parameters Presented in Section 3.3

Symbol	Long name	Description
${\langle c_{\infty }\rangle }^{1/2}$	Total fine‐scale variability	Average total fine‐scale (<250 km) variability estimated from the sill (where semivariance levels off at the decorrelation length) of spherical fits to the semivariogram
${\langle c_{s}\rangle }^{1/2}$	Spatially coherent fine‐scale variability	Average correlated fine‐scale (<250 km) variability estimated from the partial sill of spherical fits to the semivariogram (sill—nugget)
${\langle c_{0}\rangle }^{1/2}$	Random fine‐scale variability	Average random fine‐scale (<250 km) variability estimated from the nugget (*y*‐intercept) of spherical fits to the semivariogram
$\langle a_{space}\rangle$	Geostatistical range	Decorrelation length estimated from the distance at which the slope of the spherical model fit to the semivariogram levels becomes 0

**Table 3:** Average Characteristics of Sub‐Seasonal XCO_2_ Variability; Synoptic, Synoptic Variability During the Summer Months (June, July, and August) and Total Fine‐Scale Variability ${\langle c_{\infty }\rangle }^{1/2}$ Compared by Biome

	Synoptic variability (ppm)	Synoptic variability JJA (ppm)	${\langle c_{\infty }\rangle }^{1/2}$ (ppm)
N. A. Boreal	0.96	0.98	1.14
N Pacific Temperate	0.59	0.53	0.56
W Pacific Tropical	0.32	0.32	0.51
E Pacific Tropical	0.3	0.23	0.58
N. A. Temperate	0.9	1.12	0.85
Northern Ocean	0.79	0.74	0.70
N Atlantic Temperate	0.53	0.54	0.55
Atlantic Tropical	0.29	0.23	0.53
Tropical S.A.	0.37	0.11	0.77
All	0.68	0.69	0.71

**Figure 5:**
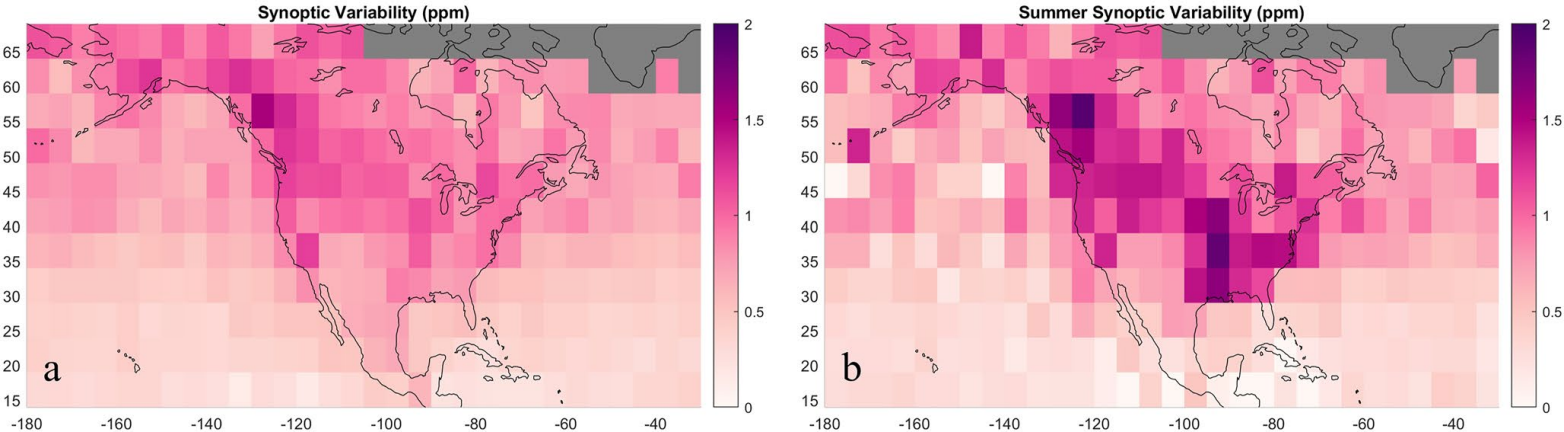
(a) Average synoptic‐scale variability. (b) Average synoptic‐scale variability during June, July, and August. We note that more scatter in summertime synoptic‐scale variability is caused by less data to compute the average; it does not indicate real features of variability changing over small scales.

Synoptic variability exhibited a strong seasonal and moderate surface‐type dependence. The greatest variability occurred during the summer months over the continental midlatitudes in a northwest‐to‐southeast pattern (Figure 5b). This pattern is similar to the gradient in mean spatial summer anomalies (Figure 3c) and average potential temperature, both illustrating mean atmospheric circulation. Outside of the summer, synoptic variability is lower on average and more uniform across the domain, decreasing into the fall and reaching a minimum over both the continent and ocean during the winter and spring. On average, summertime synoptic variability was 1.0 ppm for dominantly continental bins compared to dominantly marine bins, which were 0.5 ppm on average. The land‐ocean difference was most pronounced in the tropics. Synoptic variability in mixed surface type coastal bins was typically 0.2–0.5 ppm greater when computed using observations over land versus when we only used observations over water. Though smaller in magnitude, we still observe regional patterns of increased variability in soundings over water coastal bins, supporting they are not entirely driven by a land‐ocean bias.

Our results support findings from Keppel‐Aleks et al. (2011) that XCO_2_ variations on the atmospheric synoptic‐scale are driven by disturbances of continental‐scale XCO_2_ gradients rather than underlying surface flux variability. During the summer, great synoptic‐scale XCO_2_ variability exceeding 2 ppm occurs at the boundary of southern CO_2_‐enriched and northern CO_2_‐depleted air (refer to the asymmetrical northwest‐southeast spatial XCO_2_ gradient of ∼4 ppm in Figure 3c). The location of increased variability correlates with the mean gradient in potential temperature *θ* at 700 hPa (Figure 4b), above which XCO_2_‐depleted air caused by growing season drawdown follows large‐scale atmospheric circulation patterns. Synoptic advection across the pronounced XCO_2_ gradient creates a northwest‐southeast trending band of high synoptic variability due to the difference in XCO_2_ concentrations on either side of the large‐scale circulation‐driven gradient. Because greater differences between XCO_2_ to the north and south increase synoptic‐scale variability, greater synoptic‐scale variations could reflect greater carbon fluxes into the northern biosphere. Outside the midlatitudes, synoptic variability does not exhibit the same seasonality. In the high latitudes, synoptic variability is greater outside of summer months when climatological cyclone frequency is greater. In the subtropics and tropics, synoptic‐scale variability is greater around the continent where there is zonal disruption in wind direction (Figure S2 in Supporting Information S1). Differences in air from the westerlies transported south along the western side of the continent and air carried by the trade winds could drive synoptic XCO_2_ variability in this area.

#### 3.3.1 Fine‐Scale Variability in XCO_2_

Fine‐scale variability (computed as total along‐track variability occurring on spatial scales shorter than 250 km) and synoptic‐scale variability comprise total sub‐seasonal variability in XCO_2_. In the following sections, we partition the total fine‐scale variability ${\langle c_{\infty }\rangle }^{1/2}$ into two components: spatially coherent and random variability. Spatially coherent fine‐scale variability, ${\langle c_{s}\rangle }^{1/2}$ (Section 3.3.1) in XCO_2_ reflects variations driven by fine‐scale transport, flux variability, or systematic bias. Random fine‐scale variability, ${\langle c_{0}\rangle }^{1/2}$ (Section 3.3.3), reflects instrument noise. In Section 3.3.2, we quantify the average geostatistical spatial range, $\langle a_{space}\rangle ,$ the distance at which two points become independent. $\langle a_{space}\rangle$ quantifies the average length scale of mechanisms driving spatially coherent fine‐scale variability in XCO_2_.

These parameters are relevant to flux inversion because errors present in spatially coherent fine‐scale variability ${\langle c_{s}\rangle }^{1/2}$ cannot be effectively reduced by averaging multiple soundings like random fine‐scale variability ${\langle c_{0}\rangle }^{1/2}$ (noise). Fine‐scale spatially coherent variability can be substantially larger than reported sounding errors alone and the coherent mesoscale signal (Torres et al., 2019), leading to large representation errors in inverse modeling that have been shown to arise when mesoscale variations are not accurately constrained (Corbin et al., 2008). The geostatistical range $\langle a_{space}\rangle$ will inform modelers the distance at which XCO_2_ observations become independent of one another, which shapes the degrees of freedom in the computation of aggregate standard error. We present average characteristics and spatial patterns in these geostatistical metrics to help modelers understand the fine‐scale statistics of XCO_2_ and how they change across the domain or by season. At the end of Section 3.3.3, a comparison of these parameters for XCO_2_ collected in nadir or glint observation mode in five continental bins (Table 3) shows only minimal differences between the two modes. The comparison is limited to continental bins because nadir mode observations are only collected over land.

#### 3.3.2 Total Fine‐Scale Variability in XCO_2_

**Figure 6:**
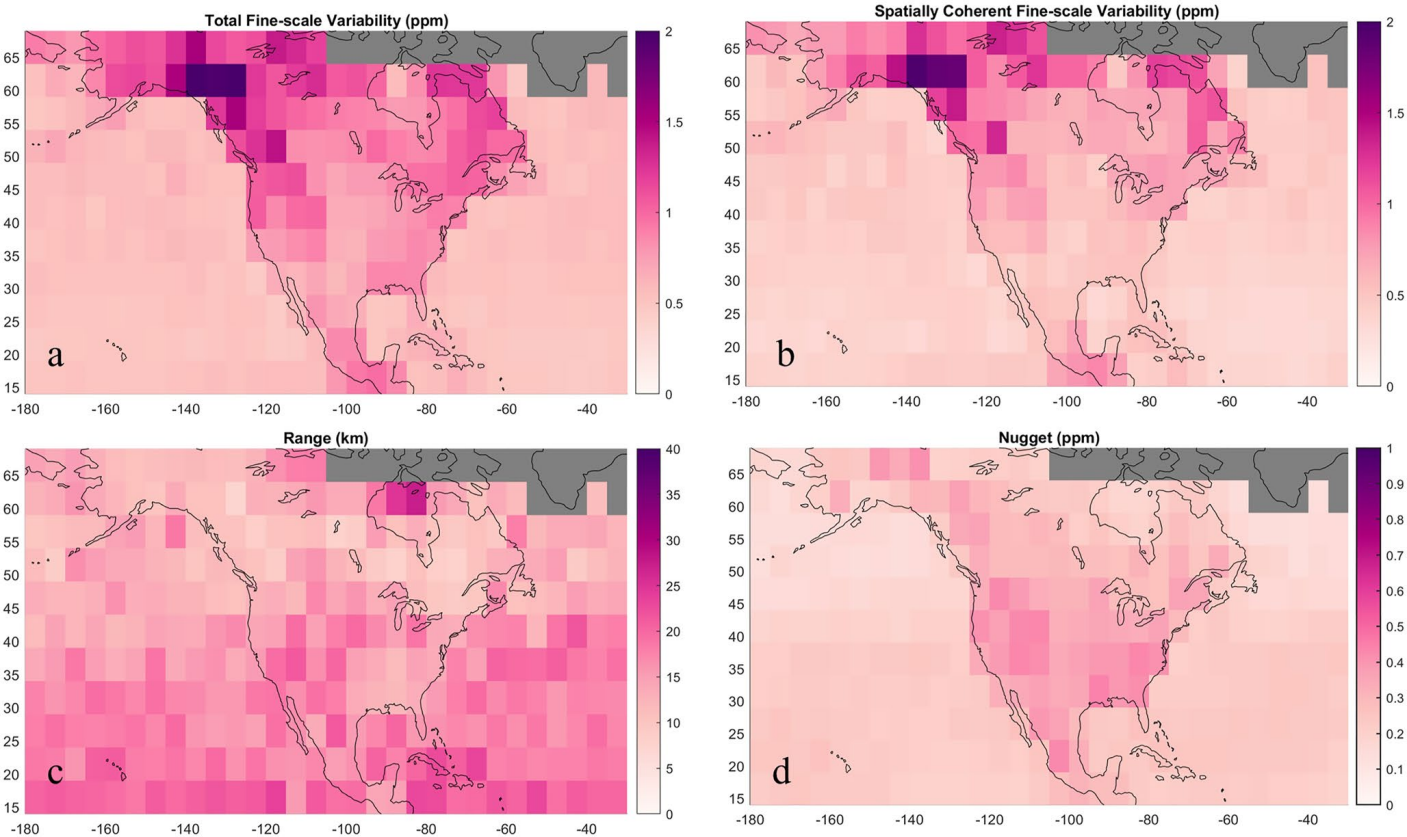
Average characteristics of fine‐scale (<250 km) variability. (a) Total fine‐scale variability ${\langle c_{\infty }\rangle }^{1/2}$, (b) spatially coherent fine‐scale variability, ${\langle c_{s}\rangle }^{1/2}$, the square root of the difference between total variance and random variance $\langle c_{\infty }\rangle -\langle c_{0}\rangle$, (c) geostatistical range $\langle a_{space}\rangle$, the separation distance at which soundings become uncorrelated, and (d) random fine‐scale variability, ${\langle c_{0}\rangle }^{1/2}$.

Average fine‐scale variability in OCO‐2 XCO_2_, ${\langle c_{\infty }\rangle }^{1/2}$, computed as total along‐track variability occurring on spatial scales shorter than 250 km, ranging from 0.5 to 2.1 ppm (Figure 6a). Compared to synoptic variability, ${\langle c_{\infty }\rangle }^{1/2}$ exhibited less seasonal variation and showed a much more robust dependence on surface type. We observed low and uniform ${\langle c_{\infty }\rangle }^{1/2}$ (generally between 0.5 and 0.7 ppm) over the ocean. ${\langle c_{\infty }\rangle }^{1/2}$ was greater (1.0 ppm on average) and more irregular over the continent. It also exhibits some of the same regional features as synoptic variability (great variability along the west coast of Canada) while lacking the large‐scale variation with latitude.

#### 3.3.3 Spatially Coherent Fine‐Scale Variability in XCO_2_

Average spatially coherent fine‐scale variability ${\langle c_{s}\rangle }^{1/2}$ in XCO_2_ ranged from 0.4 to 2.1 ppm (Figure 6b). While ${\langle c_{s}\rangle }^{1/2}$ exhibited significant spatial heterogeneity over land, ${\langle c_{s}\rangle }^{1/2}$ was robustly low and uniform over the ocean (typically 0.4–0.5 ppm). Over the continent, ${\langle c_{s}\rangle }^{1/2}$ was greatest in the boreal region (1.1 ppm on average), 0.8 ppm in the temperate region, and 0.7 ppm in the tropical region (Table 4). We observe the greatest ${\langle c_{s}\rangle }^{1/2}$ (exceeding 2 ppm) along the west coast of Canada, twice as large as the average for all continental bins (0.9 ppm). Over many bins in the middle of the continent over the Great Plains, adjacent interior lowlands west of the Great Lakes, and shrublands and desert southwestern United States, ${\langle c_{s}\rangle }^{1/2}$ was relatively low.

**Table 4:** Spatially Coherent Fine‐Scale Variability ${\langle c_{s}\rangle }^{1/2}$, Random Variability ${\langle c_{0}\rangle }^{1/2}$, and Geostatistical Range $\langle a_{space}\rangle$ by Biome

Region	${\langle c_{s}\rangle }^{1/2}$ (ppm)	${\langle c_{0}\rangle }^{1/2}$ (ppm)	$\langle a_{space}\rangle$ (km)
N. A. Boreal	1.11	0.27	12
N Pacific Temperate	0.52	0.21	16
W Pacific Tropical	0.46	0.24	21
E Pacific Tropical	0.54	0.22	20
N.A. Temperate	0.76	0.35	15
Northern Ocean	0.67	0.17	14
N Atlantic Temperate	0.49	0.23	18
Atlantic Tropical	0.48	0.22	21
N Tropical S.A.	0.72	0.26	17
All	0.66	0.24	16

Compared to synoptic‐scale variability, ${\langle c_{s}\rangle }^{1/2}$ showed much greater surface‐type dependence and far less seasonal dependence. Seasonal variations in ${\langle c_{s}\rangle }^{1/2}$ were ∼0.1 ppm for all biomes except the N.A. temperate region. Over the southeastern United States, ${\langle c_{s}\rangle }^{1/2}$ was ∼0.2 ppm greater during the summer and winter. Over the north eastern United States, ${\langle c_{s}\rangle }^{1/2}$ was ∼0.4 ppm greater during the winter. The feature of large ${\langle c_{s}\rangle }^{1/2}$ over British Columbia and the Yukon was present during spring, summer, and fall. There was insufficient data to compute an average ${\langle c_{s}\rangle }^{1/2}$ over winter months. We compared ${\langle c_{s}\rangle }^{1/2}$ by surface type in these coastal bins and found ${\langle c_{s}\rangle }^{1/2}$ over water was much lower (0.5 ppm or below) compared to over land (1–2 ppm). Our geostatistical analysis of version 9 data also revealed this feature, equal in magnitude to the v10 results. For other bins that had sufficient land and water retrievals for comparison, we found that land‐ocean differences varied geographically. Over the east coast of Canada, ${\langle c_{s}\rangle }^{1/2}$ was generally 0.5 ppm over water and 1 ppm over land. Over the tropical continent and islands, ${\langle c_{s}\rangle }^{1/2}$ was generally below 0.5 ppm when computed over water. When computed over land, ${\langle c_{s}\rangle }^{1/2}$ was closer to 1 ppm and exceeded 1.5 ppm in one bin over Hawai'i. Land and water ${\langle c_{s}\rangle }^{1/2}$ were most similar over the midlatitudes. Across the full domain, there is an average land‐ocean bias of 0.4 ppm. The average ${\langle c_{s}\rangle }^{1/2}$ for bins that have greater (>50%) land surface type fractions was 0.9 ppm, nearly twice as large as the average for bins that have greater water surface type fractions (0.5 ppm).

Across the continent, especially in the high latitudes, ${\langle c_{s}\rangle }^{1/2}$ is larger than expected for natural variations (imparted by winds or fluxes) alone (Torres et al., 2019; Worden et al., 2017). Further, we observe a distinct land‐water contrast in $c_{s}$, and while fluxes are generally smaller and less variable over the ocean, it is likely the contrast is enhanced by systematic error. Larger systematic errors can occur in XCO_2_ over land, where greater heterogeneity in surface properties like topography and albedo complicates retrieval. In particular, the exceptionally large ${\langle c_{s}\rangle }^{1/2}$ we observe in bins near the west coast of Canada was only present for land retrievals, prompting investigation into sources of regional systematic bias. We found that these bins also had the greatest average standard deviation of surface elevation, a variable provided in the sounding group of the OCO‐2 data product (Figure S3 in Supporting Information S1), suggesting a possible unresolved retrieval error related to topographic roughness. The western coast of Canada is also exceptionally cloudy, which inhibits retrieval. It is also possible the high variability is increased by a real signal related to transport as this part of the coast serves as the boundary on the atmospheric path of the jet stream between the low‐pressure zone in the North Pacific and higher pressure continent. We observed this feature in v9 XCO_2_ as well.

#### 3.3.4 Geostatistical Range of Fine‐Scale Variability in XCO_2_

The geostatistical range $\langle a_{space}\rangle$ of spatially coherent fine‐scale variability in XCO_2_ was 16 km on average for the full domain and spanned from 7 to 27 km (Figure 6c). $\langle a_{space}\rangle$ was spatially irregular across the domain, though slightly more coherent within a latitude circle over the ocean. The boreal region had the shortest $\langle a_{space}\rangle$ on average (11 km), followed by the Northern Ocean (14 km) (Table 4). The southeastern United States had relatively short $\langle a_{space}\rangle$ (under 15 km) compared to the rest of the temperate continental region. Bins over the continental tropics also had relatively short $\langle a_{space}\rangle$. Over the ocean, $\langle a_{space}\rangle$ varied from 9 to 27 km over the tropical ocean and North Pacific, respectively. $\langle a_{space}\rangle$ over the Pacific Ocean were shorter on average and more variable than over the Atlantic Ocean. $\langle a_{space}\rangle$ could be up to twice as large when weighted using proportional error as opposed to inverse error (Equation 6) but remained below 40 km and spatial patterns were consistent.

We find shorter $\langle a_{space}\rangle$ than those expected from mesoscale weather systems, further supporting that spatially coherent error is present in XCO_2_ and depresses along‐track correlation lengths. For all bins, especially in high latitudes, ranges were significantly skewed, with a peak of smaller values (<20 km) and a long tail of larger values more similar to the length scale of mesoscale systems (up to 70 km). Recent studies (Baker et al., 2022; Bell et al., 2020; Torres et al., 2019) support that spatially coherent error depresses satellite XCO_2_ ranges, particularly over land. We compared $\langle a_{space}\rangle$ computed using either majority land or water retrievals, finding $\langle a_{space}\rangle$ was significantly larger when computed over water (∼10–20 km) in the tropics and midlatitudes. While ranges over the ocean were longer than those over land on average, they were equally as short (under 15 km) over the ocean in high latitudes. This suggests a retrieval covariate over the high latitude ocean is resulting in correlated error, such as cloud cover or aerosols transported from Eurasia. There was an exception for a few bins around the Hudson Bay, where $\langle a_{space}\rangle$ was longer using water retrievals. But due to data issues, this feature may not be robust; fine‐scale statistics over this particular area should be interpreted with caution given the relatively sparse number of observations and signal‐to‐noise issues at high latitudes. Over land, we observe an inverse relationship between shorter ranges and spatially coherent variability (shorter $\langle a_{space}\rangle$ and higher ${\langle c_{s}\rangle }^{1/2}$ in the southeastern United States and western Canada). However, shorter $\langle a_{space}\rangle$ in high latitude ocean bins did not coincide with greater spatially coherent variability. Seasonal differences in $\langle a_{space}\rangle$ were all below 5 km when averaged by biome and insignificant compared to standard deviation of $\langle a_{space}\rangle$ within bins. The largest seasonal difference occurred over the midlatitudes, with $\langle a_{space}\rangle$ ∼3–5 km greater on average during summer compared to winter. Our results suggest that systematic biases are present over all times of the year across the domain.

#### 3.3.5 Random Fine‐Scale Variability in XCO_2_

Random fine‐scale variability ${\langle c_{0}\rangle }^{1/2}$ in XCO_2_ was 0.2 ppm on average for the full domain and ranged from 0.1 to 0.4 ppm (Figure 6d). For all bins, ${\langle c_{0}\rangle }^{1/2}$ was under 1 ppm, consistent with reported error from v10 OCO‐2 ACOS data product. The boreal region had lower ${\langle c_{0}\rangle }^{1/2}$ (0.27 ppm on average) compared to the continental temperate region which had the greatest ${\langle c_{0}\rangle }^{1/2}$ (0.35 ppm on average) of all regions (Table 3). We note that the average for high latitude bins does not include winter months when there are gaps in data due to insufficient light. Over the ocean, ${\langle c_{0}\rangle }^{1/2}$ was under 0.3 ppm and lower in high latitudes as well (Table 2). Compared to spatially coherent variability, ${\langle c_{s}\rangle }^{1/2}$, ${\langle c_{0}\rangle }^{1/2}$ was a generally lesser portion of total fine‐scale variability. Over the ocean, the fraction of random to total fine‐scale variability decreases with latitude from ∼30% in the subtropics to ∼18% in high latitudes. We found a robust surface‐type dependence in ${\langle c_{0}\rangle }^{1/2}$, which was typically twice as large when computed in soundings over land than ocean in mixed surface type coastal bins (0.1 ppm on average vs. 0.3 ppm). Bins that have dominantly land surface type fractions tend to have twice as large ${\langle c_{0}\rangle }^{1/2}$ compared to water bins, with the exception of some land bins in the high latitudes which have ${\langle c_{0}\rangle }^{1/2}$ that is lower and more similar to the adjacent high latitude ocean. Our results suggest a land bias of ∼0.1–0.2 ppm in random fine‐scale XCO_2_ variability, which is smaller in magnitude but more geographically robust than the potential land bias we observe in spatially coherent variability.

We observed very small seasonal differences in ${\langle c_{0}\rangle }^{1/2}$ (below 0.1 ppm) over the ocean and greater seasonal differences over the continent (the majority of land bins had ∼50% lower ${\langle c_{0}\rangle }^{1/2}$ during the winter months compared to the average across all seasons). These results are consistent with Torres et al. (2019) who reported slightly lower random variability (0.5 vs. 0.6 ppm) in 250 km high pass filtered v8 OCO‐2 XCO_2_ during winter months at Park Falls, WI and Lamont, OK. Despite low light/long path length conditions, ${\langle c_{0}\rangle }^{1/2}$ was lower during the winter at higher latitudes of the domain, supported by Torres et al. (2019) findings that random variability near their northernmost TCCON site (Bialystok, Poland) was 0.2 ppm lower during the winter (they were only able to report an average for February) compared to summer months. The greatest seasonality occurred in bins over the southeastern United States, where ${\langle c_{0}\rangle }^{1/2}$ decreased by half (∼0.2 ppm) from fall to winter. Averaged over the full continent, ${\langle c_{0}\rangle }^{1/2}$ was greatest during summer and lowest during winter. In contrast, ${\langle c_{0}\rangle }^{1/2}$ over the Great Lakes and following the Rockies was ∼0.1 ppm greater during the winter compared to other seasons and relatively large compared to the rest of the continent. The seasonal differences we observe are on the order of reported posterior v10 L2 error estimates, which were only ∼0.1 ppm over land (0.5 ppm in June vs. 0.6 ppm in December) and less than 0.1 ppm over the ocean (ranged from 0.39 to 0.45 ppm, without a clear trend by season). Seasonality in ${\langle c_{0}\rangle }^{1/2}$ may point to seasonally varying sensitivity to cloud cover or surface heterogeneity, such as vegetation and ice.

### 3.4 Relevance to Uncertainty Estimation in Inverse Modeling

Although most inverse model has a horizontal resolution sufficient to resolve synoptic scale variability, these models would still require estimates of the mean and error of fine‐scale anomalies for each orbit with valid soundings in each model grid. One possible approximation of error would be to use standard error of the fine‐scale anomalies for all soundings being averaged, *N*, assuming errors are independent for each sounding.

(8) $$\sigma_{stderr}=\sqrt{\frac{\sigma_{finescale}^{2}}{N}}=\sqrt{\frac{\langle c_{\infty }\rangle }{N}}$$

Because of the large number of soundings, N, the standard error could be substantially underestimated if not all soundings are independent. We show that soundings are not all independent but instead correlated in groups within the separation distances estimated by geostatistical ranges. The observed ranges are much shorter than typical along‐track averaging lengths used in inverse frameworks, such as across a 1° × 1° grid cell (∼110 km) or the length scale of 10 s averages (∼70 km) (as in Crowell et al., 2019 and Peiro et al., 2022). To account for the spatial correlation of the soundings in the standard error estimate, one approach would be to include separate terms for random and spatially coherent variability. For the standard error of the spatially coherent variability, an effective degrees of freedom could be computed that better represents the independence of the data, $N_{eff}$. $N_{eff}$ could be estimated using the along track averaging length, compared to the range. This assumes that each block of data equal to the size of the range is independent.

(9) $$\sigma_{stderr}=\sqrt{\frac{\langle c_{0}\rangle }{N}+\frac{\langle c_{S}\rangle }{N_{eff}}}$$

(10) $$N_{eff}=\frac{\Delta x}{\langle a_{spatial}\rangle }$$

**Figure 7:**
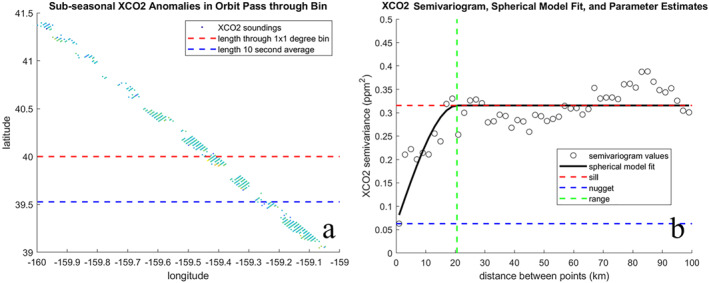
For XCO_2_ aggregated over one orbit through a 5° × 5° box, a 1° × 1° box, and over 10 s (a), 250 km high pass filtered anomalies are fit with a semivariogram to estimate standard error (b). The experimental and modeled semivariogram, estimating total fine‐scale variance $c_{\infty }$, random variance $c_{0}$, and the geostatistical range $a_{space}$. Parameter estimates were consistent across all three averaging lengths.

We compute standard error using both approaches (Equations 8 and 9) for three averaging lengths of XCO_2_; across a 5° × 5° box, a 1° × 1° box, and for a 10 s average (∼70 km). In Figure 7a, we show the three different averaging lengths over which we compute fine‐scale variability and spatial coherence for one orbit. Figure 7b shows the semivariogram and modeled $c_{\infty }$, $c_{0}$, and $a_{spatial}$ computed over one orbit through a 5° × 5° box. The modeled parameter estimates computed over a 5° × 5° box were consistent with those computed on anomalies within the 1° × 1° box and the 10 s track length shown in Figure 7a. Model estimated $a_{spatial}$ was 20.6 km, $c_{\infty }$ was 0.32 ppm^2^ and $c_{0}$ was 0.06 ppm^2^. Computed without incorporating fine‐scale spatial coherence, $\sigma_{stderr}$ was 0.03 ppm for the 5° × 5° aggregate, 0.04 for the 1° × 1° aggregate, and 0.05 ppm for the 10 s average. With spatial coherence incorporated, $\sigma_{stderr}$ was 0.14 ppm for the 5° × 5° aggregate, 0.22 ppm for the 1° × 1° aggregate, and 0.29 ppm for the 10 s average.

Using the average modeled fine‐scale variance parameters for all 5° × 5° bins (depicted in Figure 6), we compute an average $\sigma_{stderr}$ for all 5° × 5° bins using both approaches over the three averaging lengths. In Table 6, we present these results by biome. When spatial coherence is not incorporated in the standard error calculation, $\sigma_{stderr}$ is underestimated on average by 0.22 ppm for 10 s aggregates, 0.14 for 1° × 1° aggregates, and 0.07 for 5° × 5° aggregates. Standard error was largest for 10 s aggregates in the North American Boreal region (0.45 ppm) when incorporating geostatistical parameters into the computation, significantly larger than the standard error computed without geostatistical parameters, 0.14 ppm (Table 5). All other regions in the domain had 10 s aggregate standard error between 0.2 and 0.4 ppm and were underestimated by ∼0.2 ppm by the computation without geostatistical parameters. For the other aggregation lengths, standard error (ranging from less than 0.1–0.3 ppm) was typically increased by 0.1 ppm when geostatistical parameters were incorporated.

**Table 5:** Differences in Average Parameters of Fine Scale‐Variance Computed Using Either Nadir or Glint Observations for Five Bins in Different Continental Zones

Bin location	Total variance ${\langle c_{s}\rangle }^{nadir}$ − ${\langle c_{s}\rangle }^{glint}$ (ppm^2^)	Spatially coherent variance ${\langle c_{0}\rangle }^{nadir}$ − ${\langle c_{0}\rangle }^{glint}$ (ppm^2^)	Geostatistical range ${\langle a_{space}\rangle }^{nadir}$ – ${\langle a_{space}\rangle }^{glint}$ (km)
**Mexico**	−0.08	0.01	−0.03
24°–29°N 105°–100°W			
**Eastern U.S.**	−0.02	0.02	0.70
34°–39°N 85°–80°W			
**Western U.S.**	0.01	0.02	−0.68
39°–44°N 120°–115°W			
**Eastern Canada**	0.29	0.06	2.02
49°–54°N 70°–65°W			
**Western Canada**	0.06	−0.02	−1.63
49°–54°N 115°–110°W			

**Table 6:** Comparison of Standard Error in XCO_2_ Over Typical Averaging Lengths (Aggregated Over 10 s, 1° Latitude, and 5° Latitude) Computed With (Left) or Without (Right) Spatial Coherence by Biome

	$\sigma_{stderr}$ 10 s (ppm)	$\sigma_{stderr}$ 10 s (ppm)	$\sigma_{stderr}$ 1° × 1°(ppm)	$\sigma_{stderr}$ 1° × 1°(ppm)	$\sigma_{stderr}$ 5° × 5°(ppm)	$\sigma_{stderr}$ 5° × 5°(ppm)
N. A. Boreal	0.45	0.14	0.22	0.07	0.1	0.03
N Pacific Temperate	0.24	0.06	0.13	0.03	0.06	0.01
W Pacific Tropical	0.25	0.05	0.24	0.05	0.11	0.02
E Pacific Tropical	0.29	0.06	0.3	0.06	0.13	0.03
N.A. Temperate	0.35	0.09	0.25	0.06	0.11	0.03
Northern Ocean	0.3	0.08	0.17	0.05	0.08	0.02
N Atlantic Temperate	0.25	0.06	0.19	0.04	0.09	0.02
Atlantic Tropical	0.26	0.05	0.26	0.05	0.12	0.03
Tropical S.A.	0.35	0.09	0.38	0.1	0.17	0.04
All	0.30	0.08	0.19	0.05	0.09	0.02

**Figure 8:**
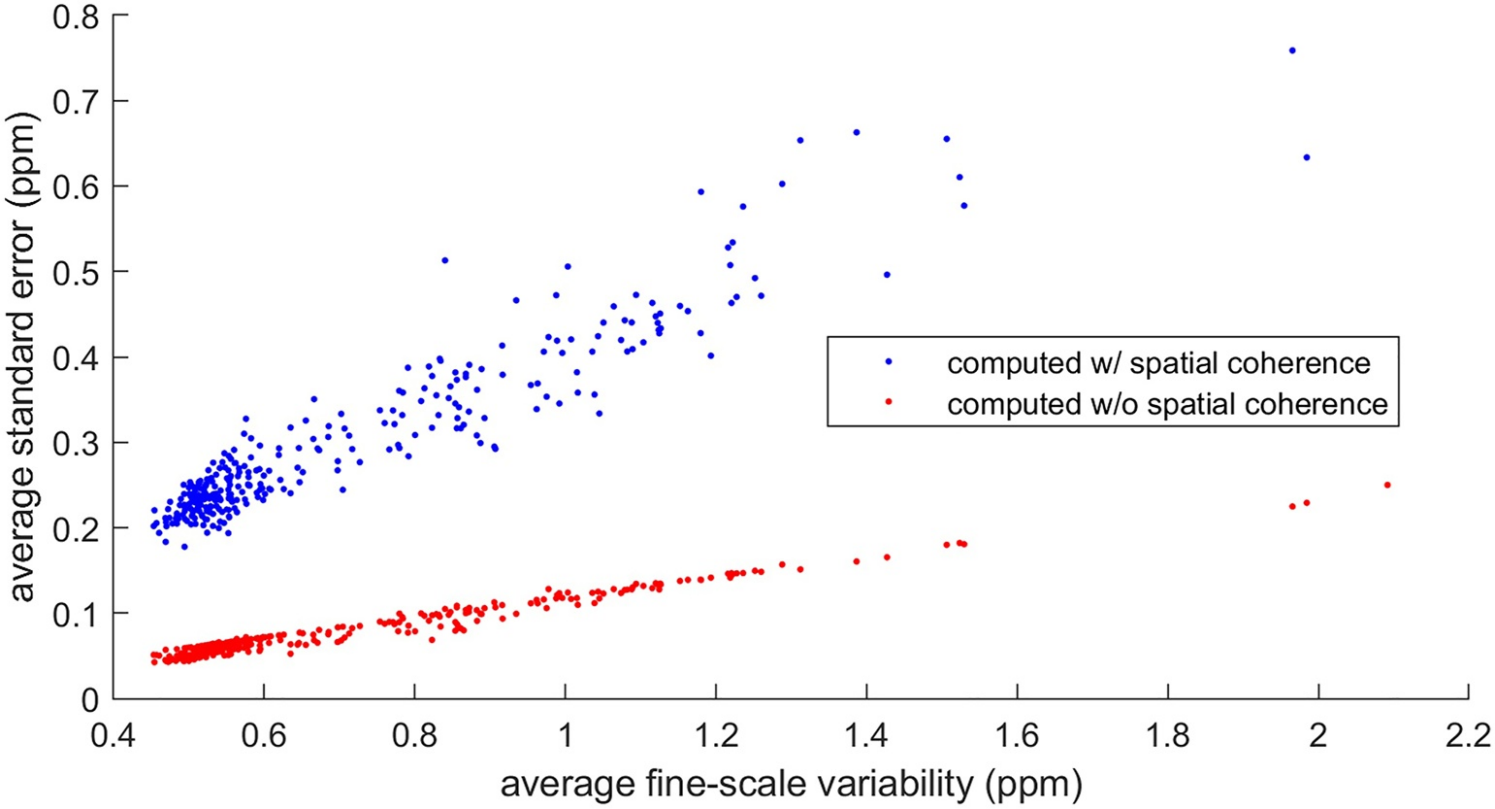
Comparison of average standard error $\sigma_{stderr}$ computed for 70 km XCO_2_ aggregates (corresponding to a typical 10 s average) for all orbit passes through 5° × 5° bins either with or without spatial coherence incorporated. Standard error scales with average fine‐scale variability $\langle c_{\infty }\rangle$.

Figure 8 shows that $\sigma_{stderr}$ exhibits a linear relationship with $\langle c_{\infty }\rangle$. For 10 s aggregates, the linear slope is 0.36 with spatial coherence incorporated and 0.12 without. Because bins with large $\langle c_{\infty }\rangle$ (>0.5 ppm) are shaped by large spatially coherent variability, $\langle c_{s}\rangle$, rather than random variability, $\langle c_{0}\rangle$, it is reasonable to assume their $\sigma_{stderr}$ is increased by correlated errors. Despite ocean bins having lower spatially coherent variability, indicating less spatially coherent bias, $\sigma_{stderr}$ is still typically underestimated by 0.1–0.2 ppm over the three averaging lengths, largely due to short geostatistical ranges.

## 4 Conclusions

We characterized the average seasonal cycle in OCO‐2 v10 XCO_2_ and partitioned sub‐seasonal XCO_2_ variability into synoptic and finer scales within 5° × 5° bins from 9‐2014 to 12‐2019 over North America and adjacent ocean basins. Using geostatistical analysis, we then quantified the magnitudes of spatially coherent and random fine‐scale (<250 km) along‐track variability. The results from this study (Mitchell, 2022) illustrate average variability on different scales and diagnose the relative influence of transport, patterns in surface fluxes, and error in the data. The primary motivation for our decomposition of variability was the present lack of understanding of fine‐scale variations and correlations in XCO_2_. Filtering out the main lower frequency modes of variability in XCO_2_ (interannual, seasonal, and synoptic scales) uncovers local patterns in XCO_2_ variability that are influenced by correlated error. While we uncovered new patterns in seasonal and synoptic‐scale XCO_2_ variability in this process, we will first discuss the implications of our fine‐scale variability characterization as this is the least‐resolved component of the XCO_2_ variance budget and presents a large barrier in estimating inverted flux uncertainty.

Geostatistical parameters indicate where fine‐scale (<250 km along‐track distance) XCO_2_ variability and correlations are driven by spatially coherent biases. Over much of the continent, particularly in high latitudes, average spatially coherent fine‐scale variability ${\langle c_{s}\rangle }^{1/2}$ reaches or exceeds 1 ppm. The large magnitude of ${\langle c_{s}\rangle }^{1/2}$ (computed as the mean from 9‐2014 to 12‐2019) cannot be reasonably explained by natural drivers like transport or local flux variability, which should produce spatially coherent variations on the order of 0.5 ppm or smaller over this short distance (Torres et al., 2019; Worden et al., 2017). In combination with length scales $\langle a_{space}\rangle$ shorter than that of mesoscale weather systems or spatial gradients driven by flux variability, we find that fine‐scale XCO_2_ over certain regions of the continent largely reflects correlated errors as opposed to real geophysical signals. Spatially coherent biases are larger over land (${\langle c_{s}\rangle }^{1/2}$ was 0.9 ppm on average) than ocean (${\langle c_{s}\rangle }^{1/2}$ was 0.5 ppm on average), but this relationship is not totally uniform. Correlated retrieval errors are widely known to be more prevalent over land, where surface properties like albedo or topography are more variable. However, we find that spatial coherent biases also affect XCO_2_ over the ocean, as shown by the short $\langle a_{space}\rangle$ values (17 km on average). Because ocean surface properties are less variable, correlated errors related to atmospheric parameters such as weather, clouds, or scatterers like aerosols may be driving the observed patterns. Errors over the ocean do not result in the large ${\langle c_{s}\rangle }^{1/2}$ as observed over land, but they do depress $\langle a_{space}\rangle$ and affect the independence of aggregated data.

Geostatistical analysis can improve estimates of XCO_2_ aggregate uncertainty and help inform how different aggregation lengths cause correlated errors to have a greater or lesser effect on flux uncertainty. We recommend modeling groups increase aggregate uncertainty for aggregates with larger $\langle a_{space}\rangle$ and greater ${\langle c_{s}\rangle }^{1/2}$ by incorporating these metrics into their error computation as in Equation 9. In 10 s along‐track XCO_2_ aggregates (∼70 km), standard error was underestimated on average by 0.22 ppm when geostatistical metrics were left out of the uncertainty computation. Standard error was also underestimated to a lesser degree in 1° × 1° aggregates (0.14 ppm greater on average using geostatistical metrics) and in 5° × 5° aggregates (0.07 ppm greater). While the effect of correlated errors on aggregate uncertainty may be considered negligible for some of the domain, aggregates in bins that have very large ${\langle c_{s}\rangle }^{1/2}$ and long $\langle a_{space}\rangle$ such as over western Canada can be underestimated by a significant amount. The greatest underestimation of uncertainty occurred when using the shortest averaging length (10 s aggregates) in these bins; standard error increased by over 0.5 ppm on average when incorporating geostatistical parameters into the uncertainty computation. Correlated errors depress $\langle a_{space}\rangle$ to distances shorter than even the shortest averaging length typically used to assimilate the data and increase uncertainty the most in those 10 s aggregates. When comparing OCO‐2 data with high‐resolution simulations of XCO_2_ such as CarbonTracker‐Lagrange or WRF forward model runs, $\langle a_{space}\rangle$ could be used to assess whether the spatial gradients in XCO_2_ are valid (observations correlated at reasonable length scales vs. too‐short scales that reflect correlated errors).

We find distinct, coherent, geostatistical characteristics in XCO_2_ over regions spanning over thousands of kilometers. For example, ${\langle c_{s}\rangle }^{1/2}$ is consistently lower across the southeastern United States and greater over the continental tropics. Over the ocean, $\langle a_{space}\rangle$ tends to decrease with increasing latitude. This indicates semivariogram analysis does not necessarily have to be performed on each individual satellite overpass assimilated into inverse models. Computing semivariogram parameters can show what areas have related geostatistical characteristics due to either surface or atmospheric properties affecting the retrieval. Modelers could then choose representative areas to assign with unique fine‐scale statistics and correlations to improve estimates of aggregate uncertainty in OCO‐2 XCO_2_ for comparison with simulated XCO_2_ in the model grid. Average geostatistical characteristics should also be computed by season; correlated errors produce features in ${\langle c_{s}\rangle }^{1/2}$ and $\langle a_{space}\rangle$ that emerge at different times of the year. Other features are present for most of the year, such as the great ${\langle c_{s}\rangle }^{1/2}$ over western Canada (we were only able to compute ${\langle c_{s}\rangle }^{1/2}$ during spring, summer, and fall, due to lacking winter observations in high latitudes). At minimum, geostatistical parameters should be computed to identify geographic locations like this with exceptionally large ${\langle c_{s}\rangle }^{1/2}$ and incorporate geostatistical metrics into error estimation.

The geostatistical parameters we computed show sharp, prominent land‐ocean differences that emerge across coastlines. Inversion studies should consider how grid cells with both land and ocean surface types such as those over a coastline will represent two distinctly different XCO_2_ distributions. In coastal bins, ${\langle c_{s}\rangle }^{1/2}$ could be up to twice as large when computed using land versus water observations. Characterizing the different fine‐scale statistics between XCO_2_ retrieved over land and water is critical for regional emissions monitoring especially over coastal urban cities. These sharp contrasts do not emerge on scales that reflect real geophysical differences, as flux or transport variations create a smoother, larger spatial gradient in total column XCO_2_. In the following paragraphs, we describe how real geophysical drivers create patterns in seasonal and synoptic‐scale XCO_2_.

The transport of large‐scale flux patterns, rather than local flux seasonality, drives the seasonal cycle in OCO‐2 XCO_2_. The most pronounced spatial gradient in XCO_2_ occurs during summer, with XCO_2_‐enriched air concentrated to the south of the jet stream and XCO_2_‐depleted air to the north caused by the hemispheric north‐south distribution of biospheric carbon uptake. XCO_2_ reaches a minimum during the fall, increases during the winter when biosphere respiration and fossil fuel emissions outweigh carbon uptake, and reaches a maximum in the spring with greatest XCO_2_ to the north. This seasonally reversing gradient is acted on by mean zonal and synoptic‐scale atmospheric circulation, driving the greatest variations in XCO_2_ on seasonal and sub‐seasonal scales. Average peak‐to‐trough seasonal cycle amplitudes in XCO_2_ were between 4.5 and 11.5 ppm and consistent with amplitudes over corresponding TCCON sites and estimated by model studies (Jacobs et al., 2021; Keppel‐Aleks et al., 2012; Sweeney et al., 2015). Bins with negligible flux seasonality experience some of the greatest seasonal XCO_2_ variability; the greatest amplitudes are concentrated in a band that extends from the Arctic to the mean path of the jet stream. Lower amplitudes are concentrated below this boundary and gradually decrease from north to south.

Seasonal XCO_2_ amplitudes reflect the Northern Hemisphere north‐south biospheric flux distribution and are spatially smoothed by large‐scale atmospheric circulation, following mean zonal flow and asymmetries. The otherwise smooth pattern in seasonal amplitudes arranged in east‐west belts is disrupted over the western continent. A distinct land‐ocean contrast manifests across the western coastline with seasonal amplitudes up to 2 ppm lower over the continent. Springtime detrended spatial means over the continent reach a lower maximum over the western continent that could result from dispersal of CO_2_‐enriched westerly air to the north and south of the coastline or a meridional transport pathway from lower latitudes up the western continent. This interesting feature prompts further scientific investigation to determine if the cause is not atmospheric circulation but instead a quasi‐stationary systematic bias related to surface type, aerosols, or an interaction between retrieval variables.

Large‐scale surface flux gradients are also responsible for XCO_2_ variability on the synoptic‐scale. Synoptic‐scale advection of XCO_2_‐depleted air from higher latitudes and XCO_2_‐enriched air from southern latitudes during the summer produced average variability over 2 ppm. Because summertime synoptic variability is greater than other seasons due to differential north‐south biologic uptake, its magnitude can be used for inferring trends in the strength of the biologic sink (Keppel‐Aleks et al., 2012; Wunch et al., 2013). These variations are sufficiently large compared to background noise and fine‐scale correlated errors in the midlatitudes to be captured by OCO‐2 (1–2 ppm). While they are greater over the continent, they extend over the midlatitude Pacific and Atlantic ocean basins as well. Summertime synoptic variability correlates with the mean gradient in potential temperature at 700 hPa, indicating that dynamical tracers can be used to validate sub‐seasonal variability in posterior XCO_2_ fields produced by inverse models. Synoptic‐scale XCO_2_ variability was also significant outside the midlatitudes and summer months, over 0.5 ppm on average across the domain. Filtering out fine‐scale variability, which can be even larger than synoptic‐scale variability at a given time and space, will help reveal the real flux and transport‐driven signals contained in synoptic‐scale variability.

Our results show spatially coherent retrieval biases still have a significant effect on the most recent version of XCO_2_ (V10) over land and ocean biomes, despite great improvements in bias correction since previous versions of the data. Because each data version are known to be affected by correlated errors and each version of the algorithm is insensitive to correlations on small (<100 km) scales, the results of this study are relevant to previous and future versions of OCO‐2 data. We observed the same feature of great ${\langle c_{s}\rangle }^{1/2}$ over British Columbia in v9 data. Future efforts to separate the influence of systematic errors from real variability would benefit from greater spatial coverage of in‐situ or aircraft high‐resolution total column measurements, particularly near coastlines and the continental areas where we found greater ${\langle c_{s}\rangle }^{1/2}$. We suggest tracking changes in geostatistical parameters with each updated version of the retrieval algorithm changes in these key areas of interest. Though the challenge of attributing error‐driven and real fine‐scale variability in OCO‐2 XCO_2_ remains, our results show that geostatistical analysis can be used to diagnose biases, improve the representation of subgrid‐scale XCO_2_, and compute more accurate estimates of aggregate uncertainty in inverse modeling. With ongoing efforts to characterize the geostatistics of dense satellite observations like OCO‐2 XCO_2_ across multiple continents and ocean basins, researchers will be better equipped to link the growing wealth of data with surface measurements and model simulations, and will be able to more accurately constrain the unique spatial and temporal patterns of surface carbon flux regions.

## Supporting information

Supporting Information S1

## Data Availability

The data produced in this study and used for this characterization of XCO_2_ variability are available at the University of Virginia Dataverse, V1, “OCO‐2 XCO_2_ Seasonal and Sub‐seasonal Variability Characterization (v10, 2014–2019)” via https://doi.org/10.18130/V3/GXOU0T (Mitchell, 2022). The retrieved Level 2 OCO‐2 XCO_2_ (version v10r) data used in this study are achived in NASA's Goddard Space Flight Center's Earth Sciences Data and Information Services Center (GES‐DISC) permanent repository (http://disc.sci.gsfc.nasa.gov/OCO-2). MATLAB (2022) and Statistics Toolbox Release 2022a software used for this research is available via (https://www.mathworks.com/products/matlab.html) © 1994–2022 The MathWorks, Inc.
