# Supplementary material for: Characterizing Average Seasonal, Synoptic, and Finer Variability in Orbiting Carbon Observatory‐2 XCO2 Across North America and Adjacent Ocean Basins
Source: J Geophys Res Atmos. 2023 Jan 27;128(3):e2022JD036696. doi: 10.1029/2022JD036696 (PMC10078313; doi:10.1029/2022JD036696)
Supplement: Supplementary file 1 — Supporting Information S1 [file JGRD-128-0-s001.pdf]

AGU PUBLICATIONS

Journal of Geophysical Research Atmospheres

Supporting Information for

Characterizing Average Seasonal, Synoptic, and Finer Variability in Orbiting Carbon Observatory-2 XCO_2 across North America and Adjacent Ocean Basins

K. A. Mitchell[1], S. C. Doney[1], and G. Keppel-Aleks[2]

[1]Environmental Sciences, University of Virginia, Charlottesville, VA, USA. [1]Environmental Sciences, University of Virginia, Charlottesville, VA, USA. [2]Climate and Space Sciences and Engineering, University of Michigan, Ann Arbor, MI, USA.

Contents of this file

Figures S1 to S3

Introduction

These figures use data provided in the OCO-2 Level 2 V10r bias-corrected XCO_2 full-physics retrieval aggregated daily files to illustrate average properties of bins used in our analysis related to wind and topography. Data associated with each sounding were averaged by bin and plotted using MATLAB version r2021a.

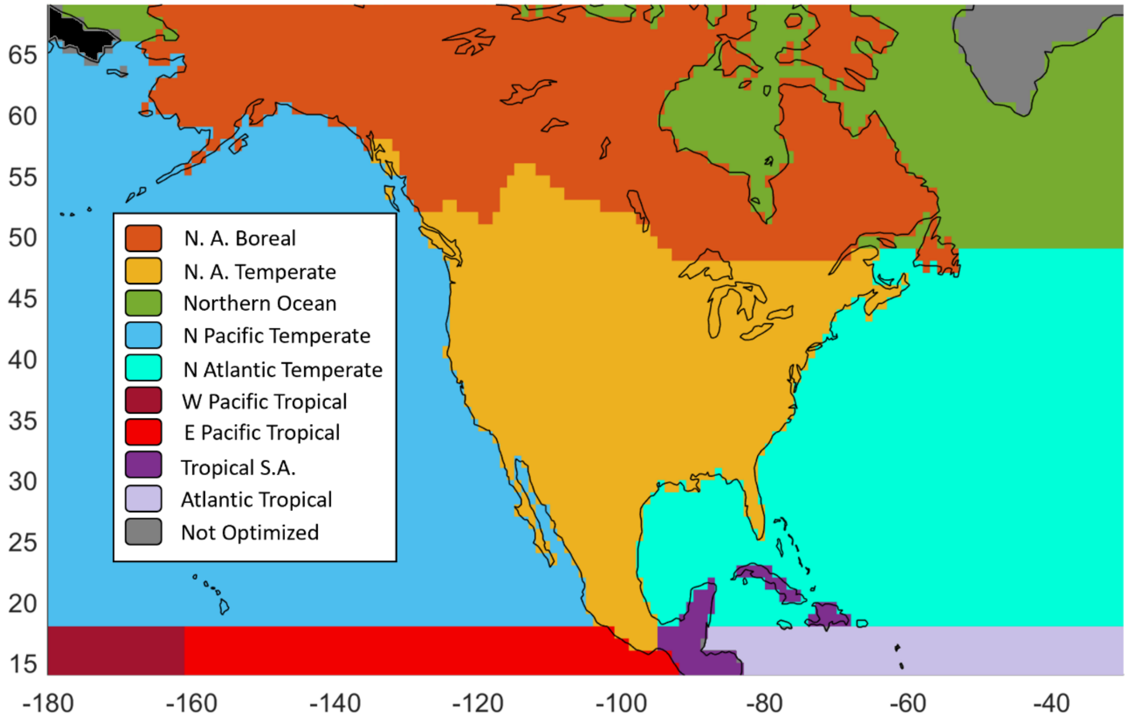

Figure S1. Map and legend of regions in our domain divided using the TransCom regional mask, as provided on the OCO-2 v10 MIP homepage at NOAA.gov. The black shaded Eurasia Boreal region was omitted from our results because we did not have enough data to represent a significant portion of this region. The gray shaded region over Greenland, TransCom region 23, is listed as "not optimized". There is not enough OCO-2 XCO_2 data in our study over this region to report results.

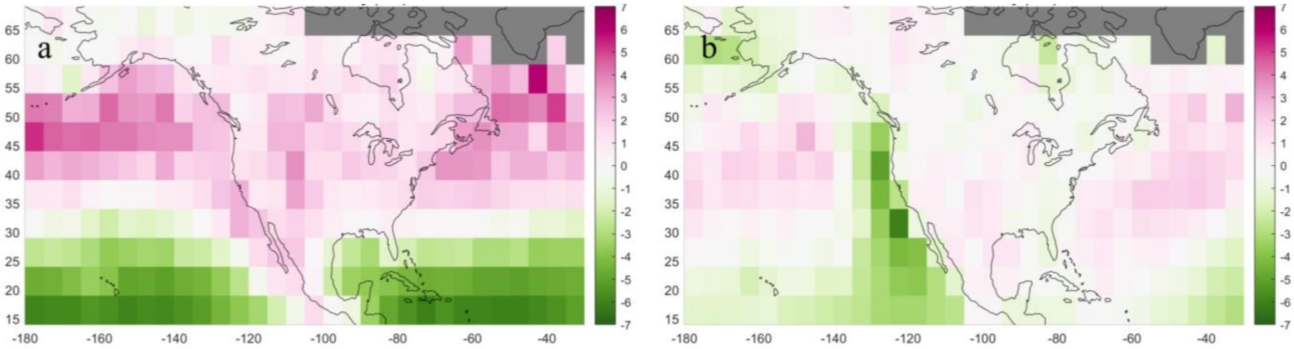

Figure S2. Average east-west (a) and north-south (b) wind velocity (m/s). This meteorological reanalysis information is provided in OCO-2 v10r Level 2 files. Positive horizontal values indicate wind is flowing to the east (is from the west)

and positive vertical values indicate wind is flowing to the north (is from the south).

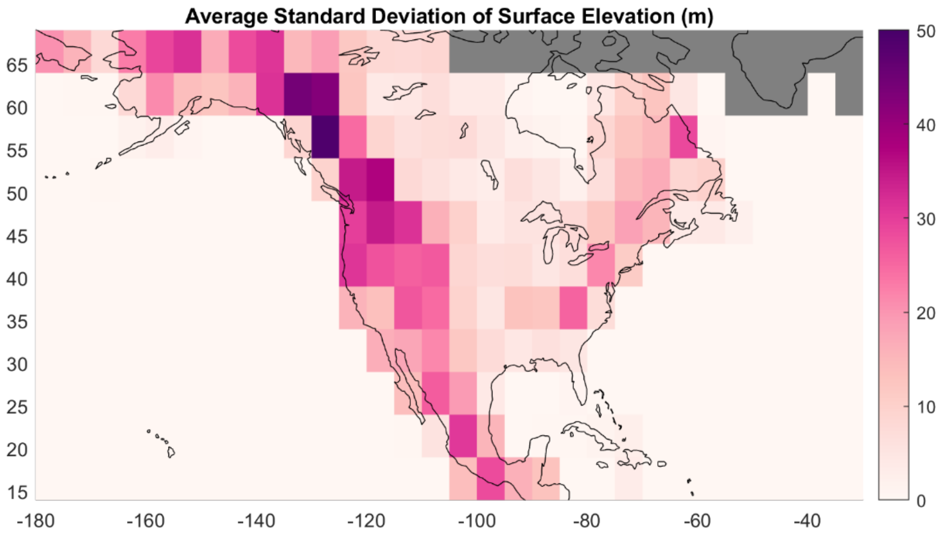

Figure S3. Average standard deviation of surface elevation in the target field of view provided in the Sounding group of OCO-2 v10r Level 2 files, illustrating topographic roughness. Bins in Western Canada with the greatest spatially coherent fine-scale variability in the domain also possess the greatest average standard deviation of surface elevation, suggesting a topography-related bias.
